# The role of exome sequencing in childhood interstitial or diffuse lung disease

**DOI:** 10.1186/s13023-022-02508-1

**Published:** 2022-09-09

**Authors:** Suzanna E. L. Temple, Gladys Ho, Bruce Bennetts, Kirsten Boggs, Nada Vidic, David Mowat, John Christodoulou, André Schultz, Thet Gayagay, Tony Roscioli, Ying Zhu, Sebastian Lunke, David Armstrong, Joanne Harrison, Nitin Kapur, Tim McDonald, Hiran Selvadurai, Andrew Tai, Zornitza Stark, Adam Jaffe

**Affiliations:** 1 Department of Clinical Genetics, Liverpool Hospital, Sydney, NSW Australia; 2 School of Women’s and Children’s Health, Faculty of Medicine and Health, UNSW, Sydney, NSW Australia; 3 Sydney Genome Diagnostics, Western Sydney Genetics Program, The Children’s Hospital at Westmead, Sydney, NSW Australia; 4 Disciplines of Child and Adolescent Health and Genomic Medicine, University of Sydney, Sydney, NSW Australia; 5 Australian Genomics Health Alliance, Melbourne, VIC Australia; 6 Department of Clinical Genetics, Children’s Hospital Westmead, Sydney, NSW Australia; 7 Centre for Clinical Genetics, Sydney Children’s Hospital Randwick, Sydney, NSW Australia; 8 University of Melbourne, Melbourne, VIC Australia; 9 Victorian Clinical Genetics Services, Murdoch Children’s Research Institute, Melbourne, VIC Australia; 10 Wal-Yan Respiratory Research Centre, Telethon Kids Institute, University of Western Australia, Perth, Australia; 11 Department of Respiratory Medicine, Perth Children’s Hospital, Nedlands, WA Australia; 12 Division of Paediatrics, Faculty of Medicine, University of Western Australia, Perth, Australia; 13 Randwick Genomics Laboratory, NSW Health Pathology, Prince of Wales Hospital, Sydney, NSW Australia; 14 Neuroscience Research Australia (NeuRA), Sydney, NSW Australia; 15 Department of Paediatrics, Monash University, Clayton Rd, Clayton, VIC Australia; 16 Department of Respiratory and Sleep Medicine, Monash Children’s Hospital, Clayton Rd, Clayton, VIC Australia; 17 Department of Respiratory and Sleep Medicine, The Royal Children’s Hospital, Melbourne, VIC Australia; 18 Department of Respiratory and Sleep Medicine, Queensland Children’s Hospital, Brisbane, QLD Australia; 19 School of Medicine, University of Queensland, Brisbane, QLD Australia; 20 The Canberra Hospital, Garran, ACT Australia; 21 Children’s Hospital Westmead, Sydney, NSW Australia; 22 Paediatric Respiratory and Sleep Department, Women’s and Children’s Hospital, Adelaide, SA Australia; 23 Robinson Research Institute, University of Adelaide, Adelaide, SA Australia; 24 Department Respiratory and Sleep Medicine, Sydney Children’s Hospital, Randwick, NSW Australia

**Keywords:** Genetics, Paediatrics, Interstitial lung disease, Paediatric lung disease, Rare lung diseases

## Abstract

**Background:**

Children’s interstitial and diffuse lung disease (chILD) is a complex heterogeneous group of lung disorders. Gene panel approaches have a reported diagnostic yield of ~ 12%. No data currently exist using trio exome sequencing as the standard diagnostic modality. We assessed the diagnostic utility of using trio exome sequencing in chILD. We prospectively enrolled children meeting specified clinical criteria between 2016 and 2020 from 16 Australian hospitals. Exome sequencing was performed with analysis of an initial gene panel followed by trio exome analysis. A subset of critically ill infants underwent ultra-rapid trio exome sequencing as first-line test.

**Results:**

36 patients [median (range) age 0.34 years (0.02–11.46); 11F] were recruited from multiple States and Territories. Five patients had clinically significant likely pathogenic/pathogenic variants (*RARB, RPL15, CTCF, RFXANK, TBX4*) and one patient had a variant of uncertain significance (*VIP*) suspected to contribute to their clinical phenotype, with *VIP* being a novel gene candidate.

**Conclusions:**

Trio exomes (6/36; 16.7%) had a better diagnostic rate than gene panel (1/36; 2.8%), due to the ability to consider a broader range of underlying conditions. However, the aetiology of chILD in most cases remained undetermined, likely reflecting the interplay between low penetrant genetic and environmental factors.

**Supplementary Information:**

The online version contains supplementary material available at 10.1186/s13023-022-02508-1.

## Background

Children’s interstitial and diffuse lung disease (chILD) is a heterogeneous group of disorders encompassing over 200 disparate rare pulmonary conditions, mostly prevalent in early childhood and often requiring life-long, complex care [1–7]. The umbrella term, chILD, has a reported prevalence of 1.5–3.8 per million [1, 4, 8] Because many of the diseases with similar clinical features do not involve the interstitium, the American Thoracic Society has suggested including the term diffuse lung diseases (DLD) [5].

The causes of chILD are multifactorial and include genetic, developmental, inflammatory, infectious and environmental contributions, all on the background of a developing and growing lung. This often means that making an accurate diagnosis is challenging and requires significant diagnostic work-up including lung function testing, imaging, bronchoalveolar lavage and lung biopsy [1, 5, 9–11]. A multidisciplinary approach involving review of patients by experts in the field has improved the diagnostic accuracy [1].

Utilising genomics in the diagnosis of chILD is advocated by both the American Thoracic Society chILD Research Network (chILDRN) and European chILD (chILD-EU) collaboration [1, 5]. A major advance has been the discovery of genetic causes of chILD, including genes associated with surfactant metabolic disorders (summarised in [12]), pulmonary haemorrhage [13], and immune dysregulation [14–16]. These gene discoveries highlight the role of next generation sequencing such as whole exome sequencing (WES) and whole genome sequencing (WGS), in advancing our understanding of chILD and leading to new approaches to treatment [17].

Most respiratory physicians favour a gene panel approach directed by phenotypic presentation. This is a cheaper option with a reported diagnostic success rate of approximately 12% [1, 4]. The cost of exome sequencing analysis is rapidly declining and is likely to replace a panel approach in the future. No data currently exist on using an exome or genome-based approach as standard clinical investigation. The aim of this study was to assess the diagnostic utility of using trio (both parents and child) exome sequencing in chILD.

## Results

**Fig. 1:**
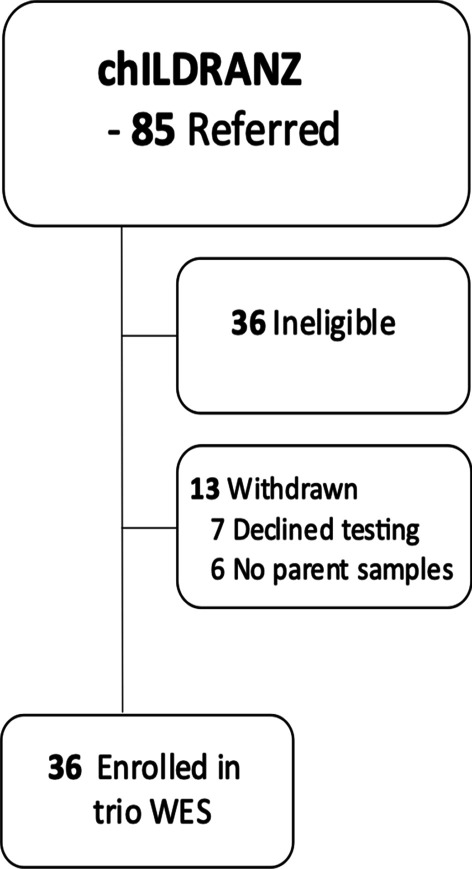
Recruitment for chILDRANZ and number enrolled in trio Whole Exome Sequencing (WES)

Eighty-five patients were referred to the chILDRANZ study. Thirty-six were excluded (42%) because they did not meet the study criteria, and 13 (26%) of those who were approved were subsequently withdrawn from the study as the family declined testing or samples from parents were not available (Fig. 1). The median age (range) of enrolled participants was 0.34 years (0.02–11.46); 30% (n = 11) were female; and patients were recruited from five Australian States and Territories (Table 1; Additional file 1: Table S1).

**Table 1:** Presenting clinical features and details of variants identified

Patient	1	2	3	4	5	6
Gender	M	F	F	M	F	F
Decimal age (years)	0.33	1.50	0.15	0.13	0.16	0.61
Gestation (wks)	41	37	34 + 1	38	40 + 6	39
Neonatal respiratory distress	−	−	+	+	+	−
Gene	*RFXANK* (OMIM#603200)	*CTCF* (OMIM#604167)	*RPL15* (OMIM#604174)	*RARB* (OMIM#180220)	*TBX4* (OMIM#601719)	*VIP* (OMIM#192320)
Condition	MHC class II deficiency, complementation group B (OMIM#209920)	Mental retardation, autosomal dominant 21 (OMIM#615502)	Diamond–Blackfan anaemia 12 (OMIM#615550)	Syndromic microphthalmia-12 (OMIM#615524)	Ischiocoxopodopatellar syndrome with or without pulmonary arterial hypertension (OMIM#147891)	
Variant	NM_003721.3 c.634C > T p.(Arg212*) Bi-parentally inherited SCV001763560.1	NM_006565) c.1699C > T p.(Arg567Trp) De novo SCV001763559.1	NM_002948.3 c.314G > T p.(Arg105Leu) De novo SCV001244976.1	NM_000965.4 c.835 T > G p.(Phe279Val) De novo SCV001245030.1	NM_018488.3 c.292C > G p.[(Pro98Ala)] Maternally inherited SUB10133140	NM_003381.3 c[107 + 1G > C] [ =]p.[?]) De novo Novel gene
Interpretation	Pathogenic	Pathogenic	Likely pathogenic	Likely pathogenic	Likely pathogenic	Variant of uncertain significance
Inheritance	Autosomal recessive	Autosomal dominant	Autosomal dominant	Autosomal dominant	Autosomal dominant	Autosomal dominant
*Clinical*						
Tachypnoea	+	−	+	+	+	+
Dyspnoea at rest	+	−	+	+	+	+
Cough	−	+	n/a	n/a	−	−
Hypoxia	+	−	+	+	+	+
Wheeze	−	+	n/a	n/a	−	−
Crackles	−	+	n/a	n/a	−	−
Recurrent infections	−	+	n/a	n/a	−	−
Pulmonary arterial hypertension	−	−	+	+	+	−
CT-chest	Ground glass changes	Air-trapping in bilateral posterior lower lobes. Posterior bilateral patchy collapse, minor bronchiectasis in bilateral lower zones	n/a	Interstitial changes	Ground glass changes	Ground glass changes
Echocardio-gram	+	−	Pulmonary stenosis	n/a	Pulmonary arterial hypertension, patent ductus arteriosus, small atrial septal defect/patent foramen ovale	−
Broncho-alveolar lavage	+Human rhinovirus	−	n/a	n/a	−	−
Biopsy	+	−	n/a	n/a	−	−
Other phenotypic features	Low IgA, IgM, IgG, T and B subsets Low pneumococcal, *Haemophilus influenzae* and diphtheria serology	Failure to thrive and gross motor delay	Intrauterine growth restriction, pulmonary stenosis, distinctive facial features (depressed nasal bridge, anteverted nares, long philtrum, narrow mouth, micrognathia), bilateral single transverse palmar crease	Diaphragmatic eventration, distinctive facial features, microphthalmia on MRI	Neonatal pneumothoraces CT angiogram demonstrated a large main pulmonary artery with hypoplastic pulmonary branch arteries consistent with PAH	Failure to thrive

Five of the 36 patients enrolled were found to have pathogenic or likely pathogenic variants (*CTCF, RFXANK, RPL15, RARB, TBX4*), and one patient had a variant of uncertain significance (*VIP*) (Table 1). Only the *TBX4* variant was identified using the chILDRANZ gene panel. No patient had an incidental variant finding, unrelated to their lung phenotype.

### *RFXANK*

Patient 1, a four-month-old boy with consanguineous parents, presented with severe respiratory distress (tachypnoea, dyspnoea at rest, hypoxia) requiring intubation, ground glass changes on CT-chest and low immunoglobulins (Table 1). Given the clinical severity, an expedited trio WES (10 days) was arranged, without the chILD gene panel first being performed. A homozygous previously reported pathogenic variant [18] was identified in the gene encoding the *Regulatory Factor X-Ankyrin repeat containing protein (RFXANK)*, with both parents being heterozygous for the variant (Table 1). Pathogenic variants in this gene cause major histocompatibility complex (MHC) class -II deficiency, also known as bare lymphocyte syndrome type II (OMIM# 209920), which is a rare immunological autosomal recessive disorder resulting from the absence of MHC class II molecules on the surface of immune cells. Individuals with biallelic variants in the *RFXANK* gene have early onset and severe recurrent respiratory and gastrointestinal infections [19].

Patient 1 underwent a lung biopsy while awaiting genetic results, which, given his severe clinical state, carried a high risk of operative morbidity. This case highlighted that critically ill patients benefit from rapid, comprehensive testing to avoid delays and had he received his genetic diagnosis earlier he would have likely avoided the need for a lung biopsy.

### *CTCF*

Patient 2 was an 18-month-old girl who presented with recurrent chest infections (associated with cough, wheeze and crackles), along with developmental delay and failure to thrive (Table 1). At the time of study referral, the treating physician considered that the mild bronchiectasis on CT-chest was insufficient to account for her respiratory symptoms and was concerned that there was an additional underlying diffuse lung process.

Trio exome analysis identified a de novo previously reported pathogenic variant in the gene encoding the CCCTC-binding factor (*CTCF*; OMIM# 604167). The CTCF protein uses various combinations of its 11 zinc fingers to recognise a variety of unrelated DNA sequences and can function as a transcriptional insulator, repressor, or activator, depending on the context of the binding site [20]. Variants in the *CTCF* gene have been associated with intellectual disability of varying severity, feeding difficulties and below normal head circumference and/or body height (SCV000491326.2; SCV001140119.1 [21]).

There has been no previous lung phenotype associated with *CTCF* variants. The respiratory symptoms observed in patient 2 were likely secondary to developmental delay and hypotonia which resulted in aspiration and mild bronchiectasis. At three years old, following PEG insertion, her respiratory symptoms had resolved.

### *RPL15*

Patient 3 was an eight-week-old girl who developed respiratory failure (tachypnoea, dyspnoea at rest, and hypoxia) and pulmonary arterial hypertension (PAH) and was noted to have distinctive facial features (Table 1). A de novo likely pathogenic variant was identified in the gene encoding the Ribosomal Protein L15 (RPL15; OMIM# 604174). Variants in the *RPL15* gene have been associated with Diamond–Blackfan anaemia 12 [22]. There are no reports of variants in the *RPL15* gene being associated with severe respiratory distress. While the finding was unexpected, the infant’s subsequent clinical course was consistent with Diamond–Blackfan anaemia.

### *RARB*

Patient 4 was a seven-week-old boy who following development of respiratory failure (tachypnoea, dyspnoea at rest, and hypoxia), was noted to have a diaphragmatic eventration in addition to PAH, and distinctive facial features including microphthalmia. Trio exome analysis identified a de novo likely pathogenic variant in the gene encoding Retinoic Acid Receptor Beta protein (RARB; OMIM# 180220). This protein binds retinoic acid and mediates cellular signalling required for the development of several organs, including the eye, heart, diaphragm, lungs and limbs [23]. Variants in the *RARB* gene have been identified with syndromic microphalmia-12 which is associated with diaphragmatic, cardiac and lung abnormalities [24, 25].

### *TBX4*

Patient 5 was a two-month-old girl who had respiratory distress (tachypnoea, dyspnoea at rest, and hypoxia) within minutes after birth requiring ventilation and developed bilateral pneumothoraces (Table 1). A CT angiogram demonstrated PAH. A maternally inherited heterozygous likely pathogenic variant was identified in the gene encoding the T-box transcription factor 4 (TBX4; OMIM# 601719). The *TBX4* gene is one of only a few genes implicated as contributing to developmental lung diseases [7]. Heterozygous variants in *TBX4* were originally associated with the autosomal dominant ischiocoxopodopatellar syndrome (ICPPS; OMIM 147891) or small patella syndrome. *TBX4* heterozygous variants have subsequently been identified as being commonly associated with childhood-onset PAH, as present in patient 5 [26–29]. There is reported variable expression with parents carrying the *TBX4* variant being asymptomatic for PAH but symptomatic for bone abnormalities associated with *TBX4* variants. Both patient 5 and her mother have the typical *TBX4* associated bone anomalies. Her mother does not have a lung phenotype. *TBX4* variants have also been reported in children with severe pulmonary hypoplasia [29, 30].

### *VIP*

Patient 6 was a three-month-old female with tachypnoea, dyspnoea at rest and hypoxia with failure to thrive (Table 1). Trio exome analysis identified a de novo heterozygous variant (c.[107 + 1G > C]; p.[?]) in the gene encoding the vasoactive intestinal peptide (VIP; OMIM# 192320). Vasoactive intestinal peptide (VIP) is a member of the glucagon-secretin family and exhibits a wide range of biologic actions including relaxation of smooth muscle [31].

VIP is abundantly expressed in the pulmonary vasculature [32]. VIP deficiency has been suggested to be involved in the development of pulmonary hypertension [33], with VIP a known neuropeptide causing relaxation of pulmonary vascular smooth muscle cells [34]. A knockout of the *VIP* gene leads to hemodynamic and histomorphological features of PAH in mice, whereas supplementation of VIP by subcutaneous application partially reversed these changes [35]. However, *VIP* is not currently considered a Mendelian disease gene and no monogenic cause for PAH has been identified in the *VIP* gene.

This splice variant has not previously been reported in the ClinVar database or in published literature and is classified as a VOUS as definitive evidence linking this gene with interstitial lung disease has not been identified. Functional investigations and ongoing review of the patient’s progress may help to further clarify the significance of this variant.

## Discussion

In our study, trio WES identified five pathogenic variants and one VOUS in 36 children with suspected chILD. Only one of these five pathogenic variants were identified using the curated chILD 75 gene panel, including disorders of surfactant. This study highlighted that lung disease is a common presentation of a number of diverse pathologies in childhood, not just primary lung disease, and a broad approach such as exome sequencing, is indicated when genetic testing is considered in the diagnostic work up of suspected chILD both in the acute and chronic disease setting. In addition, our study supports others [36] that show a higher diagnostic yield in acutely critically unwell infants with five out of six of the chILD patients with a diagnosis being four months or younger.

We did not identify any children with a surfactant disorder which was surprising. In our previous retrospective review of the chILD experience in Australia and New Zealand, genetic studies were performed for disorders of surfactant metabolism only and a diagnosis was made in 12% of patients, similar to that reported by the EU-chILD network [1]. We would therefore have expected to identify approximately five children with surfactant deficiency, but we identified six children with a broad range of other syndromic genetic disorders instead. We contacted all Australian genetic laboratories and all respiratory paediatricians and established that only five children had been tested in other laboratories outside Australia during the study period. None had a genetic surfactant disorder. Thus, the absence of this diagnosis in our cohort is most likely due to chance.

The increasing integration of genetic testing within clinical care has highlighted the need for a multi-disciplinary approach, particularly utilising the expertise of clinical geneticists and genetic counsellors in patient selection, pre-test counselling, and data interpretation. This involvement is critical with the use of trio exome (and genome) sequencing, which has the possibility of identifying variants of uncertain significance and incidental findings, which can have implications for not only the child but the child’s family. Patient 1 highlights the need for ongoing involvement of genetic services as parents who are both heterozygous carriers for an autosomal recessive condition will likely require genetic counselling to discuss reproductive options. We believe that our chILDRANZ 75 gene panel is the most comprehensive panel to date and may be useful as a curated resource for other groups to guide initial WES/WGS analysis. Only the *TBX4* variant in our study was identified using the chILD gene panel, emphasizing the need to adopter broader approaches to test requisition and analysis to maximise the diagnostic benefits of genomic data.

One limitation of our study is that our sample size was small, reflecting the low prevalence consistent with a very rare disease in a national population of only approximately five million children. The families who did not consent to be involved in the study were a mixture of active and passive decliners. There were also several families where both parents were unavailable. However, a strength is that we were able to capture most eligible children in Australia during the period of the study due to established national research and clinical networks.

Overall, our genetic diagnostic rate for likely pathogenic/pathogenic variants was 14% (5/36). While there currently remains an increased cost of trio WES compared to a targeted gene panel, although this difference continues to reduce, the benefit of a trio WES approach, rather than a chILDRANZ gene panel only (which incorporates the surfactant protein gene panel), was supported by all variants being identified using trio WES, which afforded the option of broader analysis. In addition, trio WES/WGS provides the platform to discover novel candidate genes associated with chILD, such as the *VIP* gene. The limitation of WES, and additional benefits of WGS (although significantly more expensive), is its inability to analyse deep intronic variants and structural variants.

## Conclusions

It is likely that trio WES will become standard of care in the management of chILD, particularly as costs decrease and turnaround times improve. The role of WGS to further increase diagnostic yield should be explored in future studies. Finally, our study supports the notion that chILD is a complex disorder and monogenic disorders likely make up a small proportion of the approximate 200 chILD conditions, with the underlying aetiology likely a combination of multiple common variants in conjunction with environmental factors. This study, therefore, supports a trio WES/WGS approach for children with suspected interstitial lung disease and highlights the value of multi-disciplinary team approaches as well as the need for rapid turnaround times for critically ill patients.

## Methods

Human research ethics committee approval was received from Melbourne Health (HREC/16/MH251). Parents/carers provided informed consent following genetic counselling which included the possibility of incidental findings.

### Study design and participants

These studies formed part of Australian Genomics, a national research project aimed at assessing genomic testing in healthcare [37].

The chILD research, Australia and New Zealand (chILDRANZ) flagship prospectively enrolled children between January 2016 and July 2020 from 16 Australian hospitals. Children under the age of 18 years were recruited if the treating respiratory physician suspected chILD. The inclusion criteria were based on that proposed by Deutsch et al. [11]: respiratory symptoms and/or

- diffuse infiltrates on CT scanning; and/or
- abnormal pulmonary function.

Symptoms had to be present for greater than four weeks except in neonates with acute neonatal respiratory distress.

Critically ill neonates with suspected chILD were tested through the Acute Care flagship, to access ultra-rapid trio exome sequencing. Patients with more “common causes” of diffuse lung disease and “masqueraders” were excluded [5]. A multidisciplinary panel reviewed referrals to determine eligibility.

#### Registry

The Australian Genomics secure web-based REDCap database was used for standardised systematic collection of clinical data [38]. Data elements were developed from the US chILDRN registry (Robin Deterding, personal communication) and the European chILD registry (chILD-EU) (Matthias Griese, personal communication).

#### Exome sequencing, data analysis, and interpretation

Trio exome sequencing was performed at one of three nationally accredited Australian laboratories.

We first manually curated and compiled a chILD gene panel following a systematic review of the literature (Additional file 2: Table S2). Except for critically ill neonates, alignments and variant calls generated were restricted to coding regions and the canonical splice sites of the chILDRANZ panel of 75 genes (Additional file 2: Table S2) in the proband (using SoftGenetics NextGene version 2.4.1, SoftGenetics, State College, PA; GRCh37/hg19). Variants identified were then annotated using Alamut Batch (version 1.9, Interactive Biosoftware, Rouen, France). Analysis of parental carriage of any variant identified in the proband was performed. A multidisciplinary team (MDT) board reviewed all data to ascribe causality. The initial analysis of the chILDRANZ panel data had a turnaround time of approximately six weeks. Expanded trio exome analysis was then performed, with a final result available in 3–6 months. Critically ill neonates received an ultra-rapid trio exome sequencing with results available in less than five days [36].

Variants were classified according to the American College of Medical Genetics (ACMG) criteria [39]. All pathogenic and likely pathogenic variants identified in this study have been deposited in ClinVar (submission IDs: SCV001763559.1, SCV001763560.1, SCV001244976.1, SCV001245030.1, SUB10133140). The novel gene identified in this study, *VIP*, has been submitted to GeneMatcher [40].

#### Statistical methods

As this was a prospective national epidemiological study, no power calculation was required. Descriptive statistics were used for participant characteristics, referral source, and the molecular diagnostic yield.

## Supplementary Information

**Additional file 1. Table S1.** Presenting clinical features of individuals found not to have clinically significant variants.

**Additional file 2. Table S2.** chILDRANZ gene panel (see PanelApp Australia Childhood Interstitial Lung Disease V1; gene panel has been subsequently curated and updated to V2). Gene panel was compiled following a systemic review of the literature using an exhaustive query conducted in PubMed using the following terms: ‘Interstitial lung disease’[All Fields] AND ‘paediatric’ [All Fields] OR ‘child’ [All Fields] AND (‘genes’[MeSH Terms] OR ‘gene’[All Fields]) AND ‘genetics’[All Fields] OR ‘genetics’[MeSH Terms]). Genes associated with chILD were manually curated and validated through published literature.

## Data Availability

The datasets used and/or analysed during the current study are available from the corresponding author on reasonable request. Most data generated and analysed during this study are included in this published article (and its Additional files 1 and 2).

## Abbreviations

chILD: Children’s interstitial and diffuse lung disease
DLD: Diffuse lung diseases
PAH: Pulmonary arterial hypertension
VOUS: Variant of uncertain significance
WES: Whole exome sequencing
WGS: Whole genome sequencing
