# Supplementary material for: The role of exome sequencing in childhood interstitial or diffuse lung disease
Source: Orphanet J Rare Dis. 2022 Sep 9;17:350. doi: 10.1186/s13023-022-02508-1 (PMC9463757; doi:10.1186/s13023-022-02508-1)
Supplement: Supplementary file 1 — Additional file 1. Table S1. Presenting clinical features of individuals found not to have clinically significant variants. [file 13023_2022_2508_MOESM1_ESM.docx]

| **Patient** | **7** | **8** | **9** | **10** | **11** | **12** |
| --- | --- | --- | --- | --- | --- | --- |
| Gender | F | M | M | F | M | M |
| Decimal age (years) | 3.18 | 0.42 | 0.09 | 1.16 | 1.00 | 0.13 |
| Gestation  (wks) | 35 | 40 | 36.5 | 30 | 39.4 | 38 |
| Neonatal respiratory distress | - | - | + | + | - | - |
| Gene | *ABCA3*  (OMIM#601615) | - | *FLNA*  (OMIM#300017) | - | - | - |
| Condition | Surfactant metabolism dysfunction, pulmonary, 3  (OMIM#610921) | - | Wide spectrum - skeletal dysplasia, neuronal migration abnormality, cardiovascular malformation, intellectual disability, intestinal obstruction, and pulmonary disease. | - | - | - |
| Variant | NM_001089.2  c.[737C>T]; p.[Pro246Leu]  Paternally inherited  NM_001089.2  c.[3005-4C>T]; p.[?]  Maternally inherited  (Note: sibling similarly affected, both died in infancy at 6 and 7 months old to severe ILD, sibling carrier for only single *ABCA3* variant (c.[3005-4C>T]; p.[?]) | - | NM_001110556.1 c.[1060C>T];[0] p.[His354Tyr]  Maternally inherited hemizygous  (Family referred to their local Clinical Genetics service for review and investigations) | - | - | - |
| Interpretation | Both variants of  uncertain significance | - | Variant of uncertain significance | - | - | - |
| Inheritance | Autosomal  recessive | - | Autosomal dominant | - | - | - |
| Clinical |  |  |  |  |  |  |
| Tachypnoea | + | + | + | + | + | + |
| Dyspnoea  at rest | + | - | - | - | - | - |
| Cough | + | - | - | + | + | - |
| Hypoxia | + | + | + | + | + | + |
| Wheeze | - | - | - | - | - | - |
| Crackles | - | - | - | - | + | - |
| Recurrent infections | - | - | - | + | - | - |
| Pulmonary arterial hypertension | - | - | + | + | - | - |
| CT-chest | Ground glass | Ground glass | Interstitial infiltrates | Interstitial infiltrates, alveolar infiltrates, left lower lobe collapse, ill defined opacities, hyperinflation, bronchiectasis. | Small areas of very mild ground glass opacities in medial aspect of right middle lobe and lingular segment of upper left lobe. | Ground glass |
| ECHO† | + | + | + | + | + | + |
| BAL* | + | + | - | - | + | + |
| Biopsy | - | - | - | - | - | - |
| Other phenotypic features | Failure to thrive, hypothyroidism. | Failure to thrive | Trisomy 21, fetal hydrops, right pleural effusion in utero | Failure to thrive, global developmental delay, hypotonia, mild facial dysmorphic features | Gastro-oesophageal reflux | Aspiration, infection |

| **Patient** | **13** | **14** | **15** | **16** | **17** | **18** |
| --- | --- | --- | --- | --- | --- | --- |
| Gender | F | M | F | F | M | M |
| Decimal age (years) | 2.91 | 0.13 | 0.07 | 11.46 | 0.36 | 0.77 |
| Gestation  (wks) | 40 | 32 | 40 | 40 | 40 | 39 |
| Neonatal respiratory distress | - | + | + | - | - | + |
| Gene | - | - | - | - | - | - |
| Condition | - | - | - | - | - | - |
| Variant | - | - | - | - | - | - |
| Interpretation | - | - | - | - | - | - |
| Inheritance | - | - | - | - | - | - |
| Ethnicity |  |  |  |  |  |  |
| Clinical |  |  |  |  |  |  |
| Tachypnoea | + | + | - | + | + | + |
| Dyspnoea  at rest | - | - | - (exertional dyspnoea) | - | + | - |
| Cough | + | + | - | + | - | + |
| Hypoxia | + | + | + | + | + | + |
| Wheeze | + | - | - | - | - | - |
| Crackles | + | - | - | + | + | - |
| Recurrent infections | - | - | - | - | - | - |
| Pulmonary arterial hypertension | - | - | - | - | - | - |
| CT-chest | Diffuse bilateral ground glass changes, more confluent areas of opacification in the lingula, multiple sub plural lucent areas in right middle lobe and lingula and cystic changes in right anterior and apical segments, interlobular thickening, mild traction bronchial dilation. | Reticular nodular infiltrates, ground glass pattern, numerous tiny air cysts or foci of intense alveolar gas trapping. | Ground glass | Ground glass | Ground glass right middle lobe, lingular and posterior upper lobes and apical lower lobes | Ground glass, mosaic pattern |
| ECHO† | + | + | - | +  Right ventricle dilated, dyskinetic left ventricle | - | - |
| BAL* | - | - | - | + | - | - |
| Biopsy | +  Chronic lobular remodelling, variable interstitial fibrosis, pleural thickening, cystic change, smooth muscle hyperplasia, interstitial inflammation, alveolar proteinaceous material, diffuse epithelial hyperplasia, increased alveolar macrophages,  bronchiolar damage and loss.  EM‡: ultrastructure reflects the presence of bronchiolitis obliterans. Prominent lamellar bodies with areas of irregular focal density (with several opposed and others coalescing) of type II pneumocytes suggest surfactant C deficiency. | - | - | - | - | - |
| Other phenotypic features | Mouth ulcers | - | - | Chronic microcytic anaemia, large liver and spleen | Failure to thrive, thrombocytosis | Mild chest deformity |

| **Patient** | **19** | **20** | **21** | **22** | **23** | **24** |
| --- | --- | --- | --- | --- | --- | --- |
| Gender | F | M | M | M | M | M |
| Decimal age (years) | 0.81 | 2.65 | 1.21 | 0.19 | 0.03 | 0.63 |
| Gestation (wks) | 39 | 40 | 38 | 40 | 41 | 39 |
| Neonatal respiratory distress | + | - | - | - | - | - |
| Gene | - | - | - | - | - | - |
| Condition | - | - | - | - | - | - |
| Variant | - | - | - | - | - | - |
| Interpretation | - | - | - | - | - | - |
| Inheritance | - | - | - | - | - | - |
| Clinical |  |  |  |  |  |  |
| Tachypnoea | + | + | + | + | + | + |
| Dyspnoea  at rest | - | - | - (exertional dyspnoea) | - | - | + |
| Cough | - | - | + | - | - | - |
| Hypoxia | + | + | + | + | + | + |
| Wheeze | - | - | - | - | - | - |
| Crackles | + | + | + | - | - | + |
| Recurrent infections | - | - | - | - | - | - |
| Pulmonary arterial hypertension | - | - | - | - | + | - |
| CT-chest | Ground glass, mosaic pattern, focal scattered confluent densities | Reticular infiltrates, mosaic pattern | Ground glass | Ground glass | Ground glass | Ground glass, mosaic pattern, atelectasis |
| ECHO† | + | + | + | + | +  Patent foramen ovale, slightly flattened intraventricular septum, PAH11 | + |
| BAL* | - | - | +  Pneumocystis positive on initial BAL* (repeat negative), elevated -lymphocytes | - | - | + |
| Biopsy | - | - | - | - | - | - |
| Other phenotypic features | Aspiration | - | - | Poor feeding | - | - |

| **Patient** | **25** | **26** | **27** | **28** | **29** | **30** |
| --- | --- | --- | --- | --- | --- | --- |
| Gender | M | M | F | M | M | F |
| Decimal age (years) | 0.60 | 0.14 | 5.15 | 0.25 | 1.55 | 0.17 |
| Gestation  (wks) | 33 | 37 | 36 | 31 | 3.9.5 | 35.5 |
| Neonatal respiratory distress | + | - | - | + | - | + |
| Gene | - | - | - | - | - | - |
| -Condition | - | - | - | - | - | - |
| Variant | - | - | - | - | - | - |
| Interpretation | - | - | - | - | - | - |
| Inheritance | - | - | - | - | - | - |
| Clinical |  |  |  |  |  |  |
| Tachypnoea | + | - | + | + | + | + |
| Dyspnoea  at rest | - | + | -(exertional dyspnoea) | + | - | - |
| Cough | + | + | + | - | - | - |
| Hypoxia | + | + | + | + | n/a | - |
| Wheeze | - | - | - | - | - | - |
| Crackles | - | + | - | - | - | - |
| Recurrent infections | - | - | - | - | - | - |
| Pulmonary arterial hypertension | - | - | - | - | - | + |
| CT-chest | Diffuse ground glass | Bronchial wall thickening, small foci of atelectasis. | Reticular nodular infiltrates, closely resembles organising pneumonia | Not performed | Ground glass | Mild interlobular septal thickening |
| ECHO† | + | + | + | + | n/a | + |
| BAL* | +  PJP** on nasal swab and BAL* | +  Lymphocytosis | - | - | + | + |
| Biopsy | - | - | +  Non-specific interstitial pneumonia, with a more fibrosing than cellular pattern. | - | - | - |
| Other phenotypic features | - | - | Primitive neuro-ectodermal tumor from birth treated with resection and chemotherapy | Severe chronic neonatal lung disease of prematurity, pulmonary hypoplasia, dependent. | Hip dysplasia. | Hydrops fetalis, failure to thrive, recurrent viral induced wheeze |

| **Patient** | **31** | **32** | **33** | **34** | **35** | **36** |
| --- | --- | --- | --- | --- | --- | --- |
| Gender | M | F | M | M | F | M |
| Decimal age (years) | 0.05 | 0.35 | 0.03 | 0.14 | 0.02 | 0.02 |
| Gestation  (wks) | 33 | 29+3 | 41+3 | 32 | 40+5 | 38+4 |
| Neonatal respiratory distress | + | + | - | + | - | + |
| Gene | - | - | *ABCA3*  (OMIM#601615) | - | - | - |
| Condition | - | - | Surfactant metabolism dysfunction, pulmonary, 3  (OMIM#610921) | - | - | - |
| Variant | - | - | NM_001089.2  c.1408A>C  p.(Met470Leu) | - | - | - |
| Interpretation | - | - | Heterozygous variant of uncertain significance | - | - | - |
| Inheritance | - | - | Autosomal recessive | - | - | - |
| Clinical |  |  |  |  |  |  |
| Tachypnoea | - | n/a | n/a | n/a | n/a | n/a |
| Dyspnoea  at rest | - | n/a | n/a | n/a | n/a | n/a |
| Cough | - | n/a | n/a | n/a | n/a | n/a |
| Hypoxia | + | n/a | n/a | n/a | n/a | n/a |
| Wheeze | - | n/a | n/a | n/a | n/a | n/a |
| Crackles | - | n/a | n/a | n/a | n/a | n/a |
| Recurrent infections | - | n/a | n/a | n/a | n/a | n/a |
| Pulmonary arterial hypertension | - | + | + | - | + | - |
| CT-chest | Ground glass, bi-basal consolidation | Interstitial pulmonary abnormality | n/a | Interstitial pulmonary abnormality | n/a | Interstitial pulmonary abnormality |
| ECHO† | + | Perimembranous ventricular septal defect | n/a | n/a | n/a | n/a |
| BAL* | - | n/a | n/a | n/a | n/a | n/a |
| Biopsy | - | n/a | n/a | n/a | n/a | n/a |
| Other phenotypic features | Respiratory distress at birth requiring intubation and ventilation, pulmonary haemorrhage | MCDA§ twins – sister well. Nephrocalcinosis, intrauterine growth retardation | Neonatal respiratory distress.  Haemolytic anaemia, conjugated hyperbilirubinaemia |  | Resuscitation at birth. Epileptic encephalopathy | Maternal cholestasis. Pneumothorax. |

*BAL-bronchoalveolar lavage; †ECHO-echocardiogram; ‡EM-electron microscopy; §MCDA-monochorionic diamniotic; 11PAH-pulmonary arterial hypertension; **PJP-*Pneumocystis jirovecii*.
