# Supplementary material for: The role of exome sequencing in childhood interstitial or diffuse lung disease
Source: Orphanet J Rare Dis. 2022 Sep 9;17:350. doi: 10.1186/s13023-022-02508-1 (PMC9463757; doi:10.1186/s13023-022-02508-1)
Supplement: Supplementary file 2 — Additional file 2. Table S2. chILDRANZ gene panel (see PanelApp Australia Childhood Interstitial Lung Disease V1; gene panel has been subsequently curated and updated to V2). Gene panel was compiled following a systemic review of the literature using an exhaustive query conducted in PubMed using the following terms: ‘Interstitial lung disease’[All Fields] AND ‘paediatric’ [All Fields] OR ‘child’ [All Fields] AND (‘genes’[MeSH Terms] OR ‘gene’[All Fields]) AND ‘genetics’[All Fields] OR ‘genetics’[MeSH Terms]). Genes associated with chILD were manually curated and validated through published literature. [file 13023_2022_2508_MOESM2_ESM.docx]

**Supplementary Table 2:**

| **Gene** | **Name** | **OMIM** | **Chromo** | **Inheritance** | **Lung Phenotype** | **Other Features** | **Presentation Age** | **Refs** |
| --- | --- | --- | --- | --- | --- | --- | --- | --- |
| **Genes involved in surfactant pathway** | | | | | |  |  |  |
| *ABCA3* | ATP-BINDING CASSETTE, SUBFAMILY A, MEMBER 3 | [601615](https://www.omim.org/entry/601615?search=601615&highlight=601615) | 16p13.3 | AR | Neonatal respiratory distress syndrome/ ILD | - | Neonates / Infants /Childhood | (1, 2) |
| *SFTPA1* | SURFACTANT, PULMONARY-ASSOCIATED PROTEIN A1 | [178630](https://www.omim.org/entry/178630?search=SFTPA1&highlight=sftpa1) | 10q22.3 | AD/AR | Pulmonary fibrosis/interstitial pneumonias | - | Infant | (1, 2) |
| *SFTPB* | SURFACTANT, PULMONARY-ASSOCIATED PROTEIN B | [178640](https://www.omim.org/entry/178640?search=SFTPB&highlight=sftpb) | 2p11.2 | AR | Neonatal severe respiratory distress syndrome | - | Neonates/  Infant | (1, 2) |
| *SFTPC* | SURFACTANT, PULMONARY-ASSOCIATED PROTEIN C | [178620](https://www.omim.org/entry/178620?search=SFTPc&highlight=sftpc) | 8p21.3 | AD | ILD | - | Infant | (1, 2) |
| ***Genes causing pulmonary arterial hypertension (PAH)*** | | | | | |  |  |  |
| *BMPR2* | BONE MORPHOGENETIC PROTEIN RECEPTOR, TYPE II | [600799](https://www.omim.org/entry/600799?search=600799&highlight=600799) | 2q33-q34 | AD | PAH | - | Infant🡪  Adolescence | (3, 4)  (5, 6) |
| *SMAD9* | MOTHERS AGAINST DECAPENTAPLEGIC, DROSOPHILA, HOMOLOG OF, 9 | [603295](https://www.omim.org/entry/603295?search=SMAD9&highlight=smad9) | 13q13.3 | AD | PAH | - | Infant🡪  Adolescence | (7-9) |
| *TBX4* | T-BOX 4 | [601719](https://www.omim.org/entry/601719?search=tbx4&highlight=tbx4) | 17q23.2 | AD | PAH/lung hypoplasia | OMIM:147891 Ischiococopodpatellar syndrome | Childhood | (10, 11) (3, 12) (13, 14) |
| *BMPR1B* | BONE MORPHOGENETICS PROTEIN RECEPTOR, TYPE IB | [603248](http://omim.org/entry/603248) | 4q22.3 | AD | PAH | - | Childhood🡪  Adolescence | (8, 15) |
| *CAV1* | CAVEOLIN 1 | [601047](http://omim.org/entry/601047?search=CAV1&highlight=cav1) | 7q31.2 | AD | PAH | Congenital generalised lipodystrophy | Infant/ Childhood | (16, 17) |
| *KCNK3* | POTASSIUM CHANNEL, SUBFAMILY K, MEMBER 3 | [603220](http://omim.org/entry/603220?search=KCNK3&highlight=kcnk3) | 2p23.3 | AD/AR | PAH | - | Infant | (18)(8) |
| *ACVRL1* | ACTIVIN A RECEPTOR, TYPE II-LIKE 1 | [601284](https://www.omim.org/entry/601284?search=601284&highlight=601284) | 12q13.13 | AD | HHT**/PAH | - | Infant🡪  Adolescence | (3, 5) |
| *ENG* | ENDOGLIN | 131195 | 9q34.11 | AD | HHT**/PAH | - | Infant🡪  Adolescence | (3) |
| *STRA6* | STIMULATED BY RETINOIC ACID 6 | [610745](https://www.omim.org/entry/610745?search=stra6&highlight=stra6) | 15q24.1 | AR | Lung hypoplasia/PAH | PDAC-pulmonary hypoplasia/agenesis, diaphragmatic hernia/eventration, anophthalmia/microphthalmia, and cardiac defect- syndrome. OMIM: 601186 | Neonate | (19, 20) |
| *FOXF1* | FORKHEAD BOX F1 | [601089](https://www.omim.org/entry/601089?search=FOXF1&highlight=foxf1) | 16q24.1 | AD | Alveolar capillary dysplasia/PAH | Duodenal atresia, Hirschsprung’s; urological; cardiac defects | Neonate/ Infant | (21, 22) |
| ***Genes associated with congenital central hypoventilation syndrome (CCHS)*** | | | | | |  |  |  |
| *PHOX2B* | PAIRED-LIKE HOMEOBOX 2B | [603851](https://www.omim.org/entry/603851?search=phox2b&highlight=phox2b) | 4p13 | AD | CCHS/ RDS111 | Haddad Syndrome (CCHS and Hirschsprung)  Neuroblastoma | Neonate | (23)(24) (25, 26) |
| *RET* | REARRANGED DURING TRANSFECTION PROTOONCOGENE | [164761](https://www.omim.org/entry/164761?search=ret&highlight=ret) | 10q11.21 | AD | CCHS/ RDS111 | Multiple endocrine neoplasia type II (MEN2A: OMIM:171400; MEN2B: OMIM:162300).  HSCR (OMIM:142623)  Medullary thyroid carcinoma (OMIM:155240)  Phaeochromocytoma | Neonate | (27)(28) (29) |
| *ASCL1* | ACHAETE-SCUTE COMPLEX, DROSOPHILA, HOMOLOG OF, 1 | [100790](https://www.omim.org/entry/100790?search=100790&highlight=100790) | 12q23.2 | AD | CCHS/ RDS111 | Haddad Syndrome (CCHS and Hirschsprung) | Neonate | (30) |
| *EDN3* | ENDOTHELIN 3 | [131242](https://www.omim.org/entry/131242?search=131242&highlight=131242) | 20q13.2-q13.3 | AD | CCHS/ RDS111 | Waardenburg Syndrome Type 4B;  Hirschsprung | Neonate | (31)(32) |
| *GDNF* | GLIAL CELL LINE-DERIVED NEUROTROPHIC FACTOR | [600837](https://www.omim.org/entry/600837?search=600837&highlight=600837) | 5p13.1-p12 | AD | CCHS/ RDS111 | Hirschsprung; Growth hormone deficiency | Neonate | (27)(28) (33) |
| ***Genes associated with Hermansky-Pudlak syndrome (HPS)*** | | | | | |  |  |  |
| *AP3B1* | ADAPTOR-RELATED PROTEIN COMPLEX 3, BETA-1 SUBUNIT | [603401](https://www.omim.org/entry/603401?search=603401&highlight=603401) | 5q14.1 | AR | Pulmonary fibrosis | Hermansky–Pudlak syndrome (HPS2) – oculocutaneous albinism, increased bleeding, lysosomal storage, ID††. | Childhood | (34, 35) (36) |
| *HPS1* | HPS1 GENE | [604982](https://www.omim.org/entry/604982?search=HPS1&highlight=hps1) | 10q24.2 | AR | Pulmonary fibrosis | HPS1– oculocutaneous albinism, increased bleeding, lysosomal storage, | Childhood | (37)(35) |
| *HPS4* | HPS4 GENE | [606682](https://www.omim.org/entry/606682?search=HPS4&highlight=hps4) | 22q12.1 | AR | Pulmonary fibrosis | HPS4– oculocutaneous albinism, increased bleeding, lysosomal storage | Late adolescence | (35)(38) (39) |
| *HPS6* | HPS6 GENE | [607522](https://www.omim.org/entry/607522?search=hps6&highlight=hps6) | 10q24.32 | AR | Pulmonary fibrosis | HPS6– oculocutaneous albinism, minor bleeding, lysosomal storage | Childhood | (40) |
| ***Genes associated with Hennekam Syndrome*** | | | | | |  |  |  |
| *CCBE1* | COLLAGEN AND CALCIUM-BINDING EGF DOMAIN-CONTAINING PROTEIN 1 | [612753](https://www.omim.org/entry/612753?search=ccbe1%20acted&highlight=acted%20ccbe1) | 18q21.32 | AR | Pulmonary  lymphangiectasia | Hennekam Syndrome (OMIM: 235510) – dysmorphism, ID††, lymphedema, lymphangiectasia | Neonate | (41)(42) |
| *FAT4* | FAT TUMOR SUPPRESSOR, DROSOPHILA, HOMOLOG OF, 4 | [612411](https://www.omim.org/entry/612411?search=fat4%20form&highlight=form%20fat4) | 4q28.1 | AR | Pulmonary  lymphangiectasia | Hennekam Syndrome (OMIM: 235510) – dysmorphism, ID††, lymphedema, lymphangiectasia  Van Maldergem Syndrome 2 – tracheal anamolies, microtia, ID††, skeletal dysplasia, dysmorphic, with absence of oedema. | Neonate/  Childhood | (43)(44) |
| ***Genes associated with primary ciliary dyskinesia (PCD)*** | | | | | |  |  |  |
| *DNAH5* | DYNEIN, AXONEMAL,  HEAVY CHAIN 5 | [603335](https://www.omim.org/entry/603335?search=603335&highlight=603335) | 5p102.2 | AR | Bronchiectasis | PCD (OMIM:608644)  Kartagener syndrome  Male infertility | Infant/  Childhood | (45)(46) (47) |
| *DNAI1* | DYNEIN, AXONEMAL, INTERMEDIATE CHAIN 1 | [604366](https://www.omim.org/entry/604366?search=604366&highlight=604366) | 9p13.3 | AR | Bronchiectasis | PCD  Kartagener syndrome | Childhood | (45)(46) (47) |
| *DNAL1* | DYNEIN, AXONEMAL,  LIGHT CHAIN 1 | [610062](https://www.omim.org/entry/610062?search=610062&highlight=610062) | 14q24.3 | AR | Neonatal respiratory distress syndrome/  Bronchiectasis | PCD  Kartagener syndrome | Neonate | (45)(46) (47) |
| *DNAI2* | DYNEIN, AXONEMAL, INTERMEDIATE CHAIN 2 | [605483](https://www.omim.org/entry/605483?search=605483&highlight=605483) | 17q25 | AR | Neonatal respiratory distress syndrome/  Bronchiectasis | PCD  Kartagener syndrome | Neonate | (45)(46) (47) |
| *DNAH11* | DYNEIN, AXONEMAL,  HEAVY CHAIN 11 | [603339](https://www.omim.org/entry/603339?search=603339&highlight=603339) | 7p21 | AR | Neonatal respiratory distress syndrome/  Bronchiectasis | PCD  Kartagener syndrome | Neonate | (45)(46) (47) |
| *CCDC39* | COILED-COIL DOMAIN-CONTAINING PROTEIN 39 | [613798](https://www.omim.org/entry/613798?search=613798&highlight=613798) | 3q26.33 | AR | Bronchiectasis | PCD  Kartagener syndrome  Ivemark syndrome  Male infertility | Infant | (45)(46) (47) |
| *CCDC40* | COILED-COIL DOMAIN-CONTAINING PROTEIN 40 | [613799](https://www.omim.org/entry/613799?search=613799&highlight=613799) | 17q25.3 | AR | Bronchiectasis | PCD  Kartagener syndrome | Infant | (45)(46) (47) |
| *RSPH4A* | RADIAL SPOKE HEAD 4, CHLAMYDOMONAS, HOMOLOG OF, A | [612647](https://www.omim.org/entry/612647?search=RSPH4A&highlight=rsph4a) | 6q22.1 | AR | Bronchiectasis/ILD | PCD -ChILD11  OMIM:[612649](https://www.omim.org/entry/612649)  No situs inversus | Infant/  Childhood | (45)(46) (47) |
| *RSPH9* | RADIAL SPOKE HEAD 9, CHLAMYDOMONAS, HOMOLOG OF | [612648](https://www.omim.org/entry/612648?search=RSPH9&highlight=rsph9) | 6p21.1 | AR | Bronchiectasis/ILD | PCD- ChILD12;  OMIM: [612650](https://www.omim.org/entry/612650)  No situs inversus | Infant/  Childhood | (45)(46) (47) |
| *DNAAF1* | DYNEIN, AXONEMAL, ASSEMBLY FACTOR 1 | [613190](https://www.omim.org/entry/613190?search=613190&highlight=613190) | 16q24.1 | AR | Bronchiectasis/ILD | PCD-ChILD13, OMIM-613193  Situs inversus totalis | Childhood/  Adolescence | (45)(46) (47) |
| *DNAAF2* | DYNEIN, AXONEMAL, ASSEMBLY FACTOR 2 | [612517](https://www.omim.org/entry/612517?search=612517&highlight=612517#geneMap) | 14q21.3 | AR | Bronchiectasis/ILD | PCD-ChILD10, OMIM-612518  Situs inversus totalis | Infant/  Childhood | (45)(46) (47) |
| *DNAAF3* | DYNEIN, AXONEMAL, ASSEMBLY FACTOR 3 | [614566](http://omim.org/entry/614566?search=DNAAF3&highlight=dnaaf3) | 19q13.42 | AR | Bronchiectasis/ILD | PCD | Infant/  Childhood | (45)(46) (47) |
| *RSPH1* | RADIAL SPOKE HEAD 1, CHLAMYDOMONAS, HOMOLOG OF | [609314](https://www.omim.org/entry/609314?search=rsph1&highlight=rsph1) | 21q22.3 | AR | Bronchiectasis/ILD | PCD-24  No situs inversus  Male infertility | Childhood/  Adolescence | (45)(46) (47) |
| *PIH1D3* | PIH1 DOMAIN-CONTAINING PROTEIN 3 | [300933](http://omim.org/entry/300933?search=PIH1D3&highlight=pih1d3) | Xq22.3 | XLR | Bronchiectasis/ILD | PCD-36  Male infertility | Childhood/  Adolescence | (45)(46) (47) |
| *NME8 (TXNDC3)* | NME/NM23 FAMILY MEMBER 8 | [607421](https://www.omim.org/entry/607421?search=nme8&highlight=nme8) | 7p14.1 | AR | Bronchiectasis/ILD | PCD-ChILD6 OMIM:61085 | Childhood/  Adolescence | (45)(46) (47) |
| *RPGR* | RETINITIS PIGMENTOSA GTPase REGULATOR | [312610](https://www.omim.org/entry/312610?search=rpgr&highlight=rpgr) | Xp11.4 | XL | Bronchiectasis/ILD | PCD  Retinal dystrophy  Deafness | Childhood | (45)(46) (47) |
| ***Genes associated with telomere regulation*** | | | | | |  |  |  |
| *TINF2* | TRF1-INTERACTING NUCLEAR FACTOR 2 | [604319](https://www.omim.org/entry/604319?search=tinf2&highlight=tinf2) | 14q12 | AD | Pulmonary fibrosis | Dyskeratosis congenital (DKCA3: OMIM-613990) | Infant🡪  Adolescence | (48)(49) |
| ***Genes associated with connective tissue disorders*** | | | | | |  |  |  |
| *LTBP4* | LATENT TRANSFORMING GROWTH FACTOR-BETA-BINDING PROTEIN 4, SHORT, INCLUDED | [604710](https://www.omim.org/entry/604710?search=LTBP4&highlight=ltbp4) | 19q13.2 | AR | Emphysema | Urban-Rifkin-Davis Syndrome – cutis laxa | Infant 🡪  Adolescence | (50)(51) |
| *EFEMP2 (FBLN4)* | EGF-CONTAINING FIBULIN-LIKE EXTRACELLULAR MATRIX PROTEIN 2 | [604633](https://www.omim.org/entry/604633?search=604633&highlight=604633) | 11q13.1 | AR | Acute respiratory distress syndrome | Autosomal recessive cutis laxa type 1B (ARCL1B, OMIM: 614437) - cutis laxa, arterial, craniofacial, and respiratory involvement | Neonate/  Infant | (52)(53) |
| *ELN* | ELASTIN | [130160](https://www.omim.org/entry/130160?search=130160&highlight=130160) | 7q11.23 | AD | Emphysema | OMIM:185500 & 123700  ADCL*-Cutis laxa;  Supravalvular aortic stenosis | Late adolescence | (54)(55) (56) |
| *FBLN5* | FIBULIN 5 | [604580](https://www.omim.org/entry/604580?search=604580&highlight=604580) | 14q32.1 | AR | Emphysema | ARCL†, type IA -Cutis laxa | Infant | (57)(50) |
| ***Genes associated with immunodeficiency/autoimmunity*** | | | | | |  |  |  |
| *STAT3* | SIGNAL TRANSDUCER AND ACTIVATOR OF TRANSCRIPTION 3 | [102582](https://www.omim.org/entry/102582?search=stat3&highlight=stat3) | 17q21.2 | AD | HIES11/Job Syndrome | Skeletal, dental, connective tissue abnormalities. Dysmorphic. | Childhood/  Adolescence | (58)(59) (60) |
| *DOCK8* | DEDICATOR OF CYTOKINESIS 8 | [611432](https://www.omim.org/entry/611432?search=611432&highlight=611432) | 9p24.3 | AR | HIES11/Bronchiectasis | Cutaneous viral infections, food allergies, malignancy | Childhood/  Adolescence | (61)(62) (63) |
| *ZNF341* | ZINC FINGER PROTEIN 341 | [618269](http://omim.org/entry/618269?search=znf341&highlight=znf341) | 20q11.22 | AR | HIES11 | Skeletal, dental, connective tissue abnormalities. | Childhood | (60, 64)  (65) |
| *PGM3* | PHOSPHOGLUCOMUTASE 3 | [172100](http://omim.org/entry/172100?search=pgm3%20function&highlight=%22pgm3%20function%22%20function%20pgm3) | 6q14.1 | AR | HIES11/bronchiectasis | Developmental delay, neurological deficits, neutropaenia, skeletal abnormalities. | Childhood | (66)(60) |
| *CARD11* | CASPASE RECRUITMENT DOMAIN-CONTAINING PROTEIN 11 | [607210](http://omim.org/entry/607210?search=card11%20coiled-coil%20domain&highlight=%22card11%20coiled%20coil%20domain%22%20%22card11%20coiledcoil%20domain%22%20%22coiled%20coil%22%20card11%20coiledcoil%20domain) | 7p22.2 | AD | HIES11/bronchiectasis | Similar to DOCK8 with fewer cutaneous infections | Adolescence | (67)(60) |
| *STAT1* | SIGNAL TRANSDUCER AND ACTIVATOR OF TRANSCRIPTION 1 | 600555 | 2q32.2 | AD | Bronchiectasis | Dysmorphism | Childhood | (68)(69) (70)(71) |
| *COPA* | COATOMER PROTEIN COMPLEX, SUBUNIT ALPHA | [601924](https://www.omim.org/entry/601924?search=COPA&highlight=copa) | 1q23.2 | AD | ILD | Arthritis, renal disease | Childhood | (72)(73) |
| *OAS1* | 2-PRIME,5-PRIME-OLIGOADENYLATE SYNTHETASE 1 | [164350](https://www.omim.org/entry/164350?search=oas1&highlight=oas1) | 12q24.13 | AD | PAP§§ | Hypogammaglobinaemia | Infant | (74) |
| *GATA2* | GATA-BINDING PROTEIN 2 | [137295](https://www.omim.org/entry/137295?search=GATA2&highlight=gata2) | 3q21.3 | AD | PAP§§ | Myelodysplastic syndrome | Adolescent | (75)(76) |
| *LRBA* | LIPOPOLYSACCHARIDE-RESPONSIVE, BEIGE-LIKE ANCHOR PROTEIN | [606453](https://www.omim.org/entry/606453?search=lrba&highlight=lrba) | 4q31.3 | AR | GLILD§/bronchiectasis | Immunodysregulation polyendocrinopathy enteropathy X-linked (IPEX) -like | Childhood | (77)(78) (79)(80) (81) |
| ***Genes associated with pulmonary alveolar proteinosis*** | | | | | |  |  |  |
| *CSF2RA* | COLONY-STIMULATING FACTOR 2 RECEPTOR, ALPHA | [306250](https://www.omim.org/entry/306250?search=306250&highlight=306250) | Xp22.32 | AR | PAP§§ | OMIM-300770  Surfactant metabolism dysfunction, pulmonary 4 | Childhood | (82)(83) |
| *CSF2RB* | GRANULOCYTE-MACROPHAGE COLONY-STIMULATING FACTOR RECEPTOR, BETA | [138981](https://www.omim.org/entry/138981?search=138981&highlight=138981) | 22q12.3 | AR | PAP§§ | OMIM-614370  Surfactant metabolism dysfunction, pulmonary 5 | Childhood | (82)(84) |
| ***Other genes*** | | | | | |  |  |  |
| *TMEM173*  *(STING)* | TRANSMEMBRANE PROTEIN 173 | [612374](https://www.omim.org/entry/612374?search=TMEM173&highlight=tmem173) | 5q31.2 | AD | ILD/pulmonary fibrosis | OMIM:615934  Systemic inflammation, vasculitis, angiopathy, severe skin lesions, gangrene | Neonate🡪  Adolescence | (85)(86) (87) |
| *SOX18* | SRY-BOX 18 | [601618](https://www.omim.org/entry/601618) | 20q13.33 | AD | ILD/PAH/RDS111 | Hypotrichosis lymphedema telengectasia | Neonate | (88)(89) |
| *SLC7A7* | SOLUTE CARRIER FAMILY 7 (CATIONIC AMINO ACID TRANSPORTER, y+ SYSTEM), MEMBER 7 | [603593](https://www.omim.org/entry/603593?search=SLC7A7&highlight=slc7a7) | 14q11.2 | AR | PAP§§/ILD | Lysinuric protein intolerance – hyperammoninaemia, FTT‡, hypotonia, renal abnormalities, pancytopaenia, osteoporosis. | Infant🡪  Adolescence | (90)(91) |
| *SCNN1A* | SODIUM CHANNEL, NONVOLTAGE-GATED 1, ALPHA SUBUNIT | [600228](https://www.omim.org/entry/600228?search=SCNN1A&highlight=scnn1a) | 12p13.31 | AR | Bronchiectasis | Cystic-fibrosis like. | Childhood | (92)(93) |
| *SCNN1B* | SODIUM CHANNEL, NONVOLTAGE-GATED 1, BETA SUBUNIT | [600760](https://www.omim.org/entry/600760?search=SCNN1b&highlight=scnn1b) | 16p12.2 | AR | Bronchiectasis | Transient rise in aldosterone, renin. Elevated sweat chloride test. Pseudomonas infections. | Childhood | (92)(94) |
| *NKX2-1* | NK2 HOMEOBOX 1 | [600635](https://www.omim.org/entry/600635?search=nkx2-1&highlight=nkx21%20nkx2) | 14q13.3 | AD | RDS111/ILD | Congenital hypothyroidism | Neonate/  Childhood | (95)(96) (97) |
| *MARS1* | METHIONYL-tRNA SYNTHETASE | [156560](https://www.omim.org/entry/156560?search=mars&highlight=mars) | 12q13.3 | AR | ILD/PAP§§ | FTT‡, anaemia, liver disease, developmental delay | Infant | (98)(99) (100) |
| *FGF10* | FIBROBLAST GROWTH FACTOR 10 | [602115](https://www.omim.org/entry/602115?search=FGF10&highlight=fgf10) | 5p12 | AD | Lung hypoplasia  (similar to TBX4) | Lacrimoauriculodentodigital (LAAD) syndrome | Neonate | (101)  (102) |
| *FLNA* | FILAMIN A | [300017](https://www.omim.org/entry/300017?search=FLNA&highlight=flna) | Xq28 | XL | ILD | Patent ductus arteriosus,  periventricular nodular heterotopia | Neonate /Infant | (103)  (104)  (105) |
| *FOXC2* | FORKHEAD BOX C2 | [602402](https://www.omim.org/entry/602402?search=FOXC2&highlight=foxc2) |  | AD | Pulmonary lymphangiectasia | Lower limb lymphoedema, distichiasis, ocular issues | Neonate | (106)  (107) |
| *FOXP1* | FORKHEAD BOX P1 | [605515](https://www.omim.org/entry/605515?search=FOXP1&highlight=foxp1) | 3p13 | AD | NEHI‡‡ | Hypotonia,  Developmental delay, atrial septal defect | Infant | (108) |
| *ITGA3* | INTEGRIN, ALPHA-3 | [605025](https://www.omim.org/entry/605025?search=ITGA3&highlight=itga3) | 17q21.33 | AR | ILD | Nephrotic syndrome,  Epidermolysis bullosa | Neonate 🡪Childhood | (109)  (110)  (111) (112)  (113) |
| ***Known Syndromes*** | | | | | |  |  |  |
| *FGFR2* | FIBROBLAST GROWTH FACTOR RECEPTOR 2 | [176943](https://www.omim.org/entry/176943?search=FGFR2&highlight=fgfr2) | 10q26.13 | AD/AR | RDS111/ Acinar dysplasia | Crouzon  Apert  Antley-Bixler  Ectrodactyly | Neonate | (114) |
| *CFTR* | CYSTIC FIBROSIS TRANSMEMBRANE CONDUCTANCE REGULATOR | [602421](https://www.omim.org/entry/602421?search=602421&highlight=602421) | 7q31.2 | AR | Cystic fibrosis |  | Neonate/  Childhood | (115)  (116)  (117) |
| *NF1* | NEUROFIBROMIN 1 | [613113](https://www.omim.org/entry/613113) | 17q11.2 | AD | Diffuse lung disease | Neurofibromatosis type I | Childhood | (118) |
| *FBN1* | FIBRILLIN 1 | [134797](https://www.omim.org/entry/134797?search=134797&highlight=134797) | 15q21.1 | AD | RDS111/ Pulmonary emphysema/ pneumothoraces | OMIM:154700  Marfan Syndrome-ectopic lentis, aortic root dilatation | Neonate/  Infant | (119)  (120) |
| *HRAS* | V-HA-RAS HARVEY RAT SARCOMA VIRAL ONCOGENE HOMOLOG | [190020](https://www.omim.org/entry/190020?search=HRAS&highlight=hras) | 11p15.5 | AD | RDS111/ILD/PAH | Costello | Neonate🡪 Childhood | (121)  (122)  (123) |

*ADCL: Autosomal dominant cutis laxa; †ARCL: Autosomal recessive cutis laxa; ‡FTT: Failure to thrive; §GLILD: Granulomatous and Lymphocytic interstitial lung disease; 11HIES: Hyper-IgE syndrome; **HHT: [Hereditary haemorrhagic telangiectasia](https://encyclopedia.thefreedictionary.com/Hereditary+hemorrhagic+telangiectasia); ††ID: immunodeficiency; ‡‡NEHI: Neuroendocrine hyperplasia of infancy; §§PAP: Pulmonary alveolar proteinosis; 111RDS: Respiratory distress of the newborn.

**REFERENCES:**

1. Singh J, Jaffe A, Schultz A, Selvadurai H. Surfactant protein disorders in childhood interstitial lung disease. Eur J Pediatr 2021; 180: 2711-2721.

2. Nogee LM. Genetic causes of surfactant protein abnormalities. Curr Opin Pediatr 2019; 31: 330-339.

3. Levy M, Eyries M, Szezepanski I, Ladouceur M, Nadaud S, Bonnet D, Soubrier F. Genetic analyses in a cohort of children with pulmonary hypertension. Eur

Respir J 2016; 48: 1118-1126.

4. Soubrier F, Chung WK, Machado R, Grünig E, Aldred M, Geraci M, Loyd JE, Elliott CG, Trembath RC, Newman JH, Humbert M. Genetics and genomics of

pulmonary arterial hypertension. J Am Coll Cardiol 2013; 62: D13-21.

5. Chida A, Shintani M, Yagi H, Fujiwara M, Kojima Y, Sato H, Imamura S, Yokozawa M, Onodera N, Horigome H, Kobayashi T, Hatai Y, Nakayama T,

Fukushima H, Nishiyama M, Doi S, Ono Y, Yasukouchi S, Ichida F, Fujimoto K, Ohtsuki S, Teshima H, Kawano T, Nomura Y, Gu H, Ishiwata T, Furutani

Y, Inai K, Saji T, Matsuoka R, Nonoyama S, Nakanishi T. Outcomes of childhood pulmonary arterial hypertension in BMPR2 and ALK1 mutation

carriers. Am J Cardiol 2012; 110: 586-593.

6. Machado RD, Pauciulo MW, Thomson JR, Lane KB, Morgan NV, Wheeler L, Phillips JA, 3rd, Newman J, Williams D, Galiè N, Manes A, McNeil K, Yacoub M,

Mikhail G, Rogers P, Corris P, Humbert M, Donnai D, Martensson G, Tranebjaerg L, Loyd JE, Trembath RC, Nichols WC. BMPR2 haploinsufficiency as

the inherited molecular mechanism for primary pulmonary hypertension. Am J Hum Genet 2001; 68: 92-102.

7. Nasim MT, Ogo T, Ahmed M, Randall R, Chowdhury HM, Snape KM, Bradshaw TY, Southgate L, Lee GJ, Jackson I, Lord GM, Gibbs JS, Wilkins MR, Ohta-

Ogo K, Nakamura K, Girerd B, Coulet F, Soubrier F, Humbert M, Morrell NW, Trembath RC, Machado RD. Molecular genetic characterization of

SMAD signaling molecules in pulmonary arterial hypertension. Hum Mutat 2011; 32: 1385-1389.

8. Garcia-Rivas G, Jerjes-Sánchez C, Rodriguez D, Garcia-Pelaez J, Trevino V. A systematic review of genetic mutations in pulmonary arterial hypertension.

BMC Med Genet 2017; 18: 82.

9. Shintani M, Yagi H, Nakayama T, Saji T, Matsuoka R. A new nonsense mutation of SMAD8 associated with pulmonary arterial hypertension. J Med Genet

2009; 46: 331-337.

10. Zhu N, Gonzaga-Jauregui C, Welch CL, Ma L, Qi H, King AK, Krishnan U, Rosenzweig EB, Ivy DD, Austin ED, Hamid R, Nichols WC, Pauciulo MW, Lutz KA,

Sawle A, Reid JG, Overton JD, Baras A, Dewey F, Shen Y, Chung WK. Exome Sequencing in Children With Pulmonary Arterial Hypertension

Demonstrates Differences Compared With Adults. Circ Genom Precis Med 2018; 11: e001887.

11. Kerstjens-Frederikse WS, Bongers EM, Roofthooft MT, Leter EM, Douwes JM, Van Dijk A, Vonk-Noordegraaf A, Dijk-Bos KK, Hoefsloot LH, Hoendermis

ES, Gille JJ, Sikkema-Raddatz B, Hofstra RM, Berger RM. TBX4 mutations (small patella syndrome) are associated with childhood-onset pulmonary

arterial hypertension. J Med Genet 2013; 50: 500-506.

12. Eyries M, Montani D, Nadaud S, Girerd B, Levy M, Bourdin A, Trésorier R, Chaouat A, Cottin V, Sanfiorenzo C, Prevot G, Reynaud-Gaubert M, Dromer C,

Houeijeh A, Nguyen K, Coulet F, Bonnet D, Humbert M, Soubrier F. Widening the landscape of heritable pulmonary hypertension mutations in

paediatric and adult cases. Eur Respir J 2019; 53.

13. Szafranski P, Coban-Akdemir ZH, Rupps R, Grazioli S, Wensley D, Jhangiani SN, Popek E, Lee AF, Lupski JR, Boerkoel CF, Stankiewicz P. Phenotypic

expansion of TBX4 mutations to include acinar dysplasia of the lungs. Am J Med Genet A 2016; 170: 2440-2444.

14. Suhrie K, Pajor NM, Ahlfeld SK, Dawson DB, Dufendach KR, Kitzmiller JA, Leino D, Lombardo RC, Smolarek TA, Rathbun PA, Whitsett JA, Towe C,

Wikenheiser-Brokamp KA. Neonatal Lung Disease Associated with TBX4 Mutations. J Pediatr 2019; 206: 286-292 e281.

15. Chida A, Shintani M, Nakayama T, Furutani Y, Hayama E, Inai K, Saji T, Nonoyama S, Nakanishi T. Missense mutations of the BMPR1B (ALK6) gene in

childhood idiopathic pulmonary arterial hypertension. Circ J 2012; 76: 1501-1508.

16. Han B, Copeland CA, Kawano Y, Rosenzweig EB, Austin ED, Shahmirzadi L, Tang S, Raghunathan K, Chung WK, Kenworthy AK. Characterization of a

caveolin-1 mutation associated with both pulmonary arterial hypertension and congenital generalized lipodystrophy. Traffic 2016; 17: 1297-1312.

17. Austin ED, Ma L, LeDuc C, Berman Rosenzweig E, Borczuk A, Phillips JA, 3rd, Palomero T, Sumazin P, Kim HR, Talati MH, West J, Loyd JE, Chung WK.

Whole exome sequencing to identify a novel gene (caveolin-1) associated with human pulmonary arterial hypertension. Circ Cardiovasc Genet

2012; 5: 336-343.

18. Navas Tejedor P, Tenorio Castaño J, Palomino Doza J, Arias Lajara P, Gordo Trujillo G, López Meseguer M, Román Broto A, Lapunzina Abadía P, Escribano

Subía P. An homozygous mutation in KCNK3 is associated with an aggressive form of hereditary pulmonary arterial hypertension. Clin Genet 2017;

91: 453-457.

19. Andijani AA, Shajira ES, Abushaheen A, Al-Matary A. Microphthalmia Syndrome 9: Case Report of a Newborn Baby with Pulmonary Hypoplasia,

Diaphragmatic Eventration, Microphthalmia, Cardiac Defect and Severe Primary Pulmonary Hypertension. Am J Case Rep 2019; 20: 354-360.

20. Pasutto F, Flinter F, Rauch A, Reis A. Novel STRA6 null mutations in the original family described with Matthew-Wood syndrome. Am J Med Genet A

2018; 176: 134-138.

21. Stankiewicz P, Sen P, Bhatt SS, Storer M, Xia Z, Bejjani BA, Ou Z, Wiszniewska J, Driscoll DJ, Maisenbacher MK, Bolivar J, Bauer M, Zackai EH, McDonald-

McGinn D, Nowaczyk MM, Murray M, Hustead V, Mascotti K, Schultz R, Hallam L, McRae D, Nicholson AG, Newbury R, Durham-O'Donnell J, Knight

G, Kini U, Shaikh TH, Martin V, Tyreman M, Simonic I, Willatt L, Paterson J, Mehta S, Rajan D, Fitzgerald T, Gribble S, Prigmore E, Patel A, Shaffer LG,

Carter NP, Cheung SW, Langston C, Shaw-Smith C. Genomic and genic deletions of the FOX gene cluster on 16q24.1 and inactivating mutations of

FOXF1 cause alveolar capillary dysplasia and other malformations. Am J Hum Genet 2009; 84: 780-791.

22. Sen P, Yang Y, Navarro C, Silva I, Szafranski P, Kolodziejska KE, Dharmadhikari AV, Mostafa H, Kozakewich H, Kearney D, Cahill JB, Whitt M, Bilic M,

Margraf L, Charles A, Goldblatt J, Gibson K, Lantz PE, Garvin AJ, Petty J, Kiblawi Z, Zuppan C, McConkie-Rosell A, McDonald MT, Peterson-Carmichael

SL, Gaede JT, Shivanna B, Schady D, Friedlich PS, Hays SR, Palafoll IV, Siebers-Renelt U, Bohring A, Finn LS, Siebert JR, Galambos C, Nguyen L, Riley

M, Chassaing N, Vigouroux A, Rocha G, Fernandes S, Brumbaugh J, Roberts K, Ho-Ming L, Lo IF, Lam S, Gerychova R, Jezova M, Valaskova I, Fellmann

F, Afshar K, Giannoni E, Muhlethaler V, Liang J, Beckmann JS, Lioy J, Deshmukh H, Srinivasan L, Swarr DT, Sloman M, Shaw-Smith C, van Loon RL,

Hagman C, Sznajer Y, Barrea C, Galant C, Detaille T, Wambach JA, Cole FS, Hamvas A, Prince LS, Diderich KE, Brooks AS, Verdijk RM, Ravindranathan

H, Sugo E, Mowat D, Baker ML, Langston C, Welty S, Stankiewicz P. Novel FOXF1 mutations in sporadic and familial cases of alveolar capillary

dysplasia with misaligned pulmonary veins imply a role for its DNA binding domain. Hum Mutat 2013; 34: 801-811.

23. Zhou A, Rand CM, Hockney SM, Niewijk G, Reineke P, Speare V, Berry-Kravis EM, Zhou L, Jennings LJ, Yu M, Ceccherini I, Bachetti T, Pennock M, Yap KL,

Weese-Mayer DE. Paired-like homeobox gene (PHOX2B) nonpolyalanine repeat expansion mutations (NPARMs): genotype-phenotype correlation in

congenital central hypoventilation syndrome (CCHS). Genet Med 2021; 23: 1656-1663.

24. Weese-Mayer DE, Berry-Kravis EM, Ceccherini I, Keens TG, Loghmanee DA, Trang H. An official ATS clinical policy statement: Congenital central

hypoventilation syndrome: genetic basis, diagnosis, and management. Am J Respir Crit Care Med 2010; 181: 626-644.

25. Trochet D, O'Brien LM, Gozal D, Trang H, Nordenskjöld A, Laudier B, Svensson PJ, Uhrig S, Cole T, Niemann S, Munnich A, Gaultier C, Lyonnet S, Amiel J.

PHOX2B genotype allows for prediction of tumor risk in congenital central hypoventilation syndrome. Am J Hum Genet 2005; 76: 421-426.

26. Berry-Kravis EM, Zhou L, Rand CM, Weese-Mayer DE. Congenital central hypoventilation syndrome: PHOX2B mutations and phenotype. Am J Respir Crit

Care Med 2006; 174: 1139-1144.

27. Amiel J, Salomon R, Attié T, Pelet A, Trang H, Mokhtari M, Gaultier C, Munnich A, Lyonnet S. Mutations of the RET-GDNF signaling pathway in Ondine's

curse. Am J Hum Genet 1998; 62: 715-717.

28. Amiel J, Laudier B, Attié-Bitach T, Trang H, de Pontual L, Gener B, Trochet D, Etchevers H, Ray P, Simonneau M, Vekemans M, Munnich A, Gaultier C,

Lyonnet S. Polyalanine expansion and frameshift mutations of the paired-like homeobox gene PHOX2B in congenital central hypoventilation

syndrome. Nat Genet 2003; 33: 459-461.

29. Kanai M, Numakura C, Sasaki A, Shirahata E, Akaba K, Hashimoto M, Hasegawa H, Shirasawa S, Hayasaka K. Congenital central hypoventilation

syndrome: a novel mutation of the RET gene in an isolated case. Tohoku J Exp Med 2002; 196: 241-246.

30. de Pontual L, Népote V, Attié-Bitach T, Al Halabiah H, Trang H, Elghouzzi V, Levacher B, Benihoud K, Augé J, Faure C, Laudier B, Vekemans M, Munnich A,

Perricaudet M, Guillemot F, Gaultier C, Lyonnet S, Simonneau M, Amiel J. Noradrenergic neuronal development is impaired by mutation of the

proneural HASH-1 gene in congenital central hypoventilation syndrome (Ondine's curse). Hum Mol Genet 2003; 12: 3173-3180.

31. Bolk S, Angrist M, Xie J, Yanagisawa M, Silvestri JM, Weese-Mayer DE, Chakravarti A. Endothelin-3 frameshift mutation in congenital central

hypoventilation syndrome. Nat Genet 1996; 13: 395-396.

32. Weese-Mayer DE, Berry-Kravis EM, Zhou L, Maher BS, Silvestri JM, Curran ME, Marazita ML. Idiopathic congenital central hypoventilation syndrome:

analysis of genes pertinent to early autonomic nervous system embryologic development and identification of mutations in PHOX2b. Am J Med

Genet A 2003; 123a: 267-278.

33. Sasaki A, Kanai M, Kijima K, Akaba K, Hashimoto M, Hasegawa H, Otaki S, Koizumi T, Kusuda S, Ogawa Y, Tuchiya K, Yamamoto W, Nakamura T, Hayasaka

K. Molecular analysis of congenital central hypoventilation syndrome. Hum Genet 2003; 114: 22-26.

34. El-Chemaly S, Young LR. Hermansky-Pudlak Syndrome. Clin Chest Med 2016; 37: 505-511.

35. Huizing M, Scher CD, Strovel E, Fitzpatrick DL, Hartnell LM, Anikster Y, Gahl WA. Nonsense mutations in ADTB3A cause complete deficiency of the

beta3A subunit of adaptor complex-3 and severe Hermansky-Pudlak syndrome type 2. Pediatr Res 2002; 51: 150-158.

36. Shotelersuk V, Dell'Angelica EC, Hartnell L, Bonifacino JS, Gahl WA. A new variant of Hermansky-Pudlak syndrome due to mutations in a gene

responsible for vesicle formation. Am J Med 2000; 108: 423-427.

37. Kelil T, Shen J, O'Neill AC, Howard SA. Hermansky-pudlak syndrome complicated by pulmonary fibrosis: radiologic-pathologic correlation and review of

pulmonary complications. J Clin Imaging Sci 2014; 4: 59.

38. Bachli EB, Brack T, Eppler E, Stallmach T, Trüeb RM, Huizing M, Gahl WA. Hermansky-Pudlak syndrome type 4 in a patient from Sri Lanka with pulmonary

fibrosis. Am J Med Genet A 2004; 127a: 201-207.

39. Anderson PD, Huizing M, Claassen DA, White J, Gahl WA. Hermansky-Pudlak syndrome type 4 (HPS-4): clinical and molecular characteristics. Hum Genet

2003; 113: 10-17.

40. Andres O, Wiegering V, König EM, Schneider AL, Semeniak D, Stritt S, Klopocki E, Schulze H. A novel two-nucleotide deletion in HPS6 affects mepacrine

uptake and platelet dense granule secretion in a family with Hermansky-Pudlak syndrome. Pediatr Blood Cancer 2017; 64.

41. Frosk P, Chodirker B, Simard L, El-Matary W, Hanlon-Dearman A, Schwartzentruber J, Majewski J, Rockman-Greenberg C. A novel CCBE1 mutation

leading to a mild form of hennekam syndrome: case report and review of the literature. BMC Med Genet 2015; 16: 28.

42. Alders M, Hogan BM, Gjini E, Salehi F, Al-Gazali L, Hennekam EA, Holmberg EE, Mannens MM, Mulder MF, Offerhaus GJ, Prescott TE, Schroor EJ, Verheij

JB, Witte M, Zwijnenburg PJ, Vikkula M, Schulte-Merker S, Hennekam RC. Mutations in CCBE1 cause generalized lymph vessel dysplasia in humans.

Nat Genet 2009; 41: 1272-1274.

43. Alders M, Al-Gazali L, Cordeiro I, Dallapiccola B, Garavelli L, Tuysuz B, Salehi F, Haagmans MA, Mook OR, Majoie CB, Mannens MM, Hennekam RC.

Hennekam syndrome can be caused by FAT4 mutations and be allelic to Van Maldergem syndrome. Hum Genet 2014; 133: 1161-1167.

44. Al-Gazali LI, Hertecant J, Ahmed R, Khan NA, Padmanabhan R. Further delineation of Hennekam syndrome. Clin Dysmorphol 2003; 12: 227-232.

45. Horani A, Ferkol TW. Advances in the Genetics of Primary Ciliary Dyskinesia: Clinical Implications. Chest 2018; 154: 645-652.

46. Horani A, Ferkol TW. Understanding Primary Ciliary Dyskinesia and Other Ciliopathies. J Pediatr 2021; 230: 15-22.e11.

47. Lucas JS, Davis SD, Omran H, Shoemark A. Primary ciliary dyskinesia in the genomics age. Lancet Respir Med 2020; 8: 202-216.

48. Sasa GS, Ribes-Zamora A, Nelson ND, Bertuch AA. Three novel truncating TINF2 mutations causing severe dyskeratosis congenita in early childhood. Clin

Genet 2012; 81: 470-478.

49. Tsangaris E, Adams SL, Yoon G, Chitayat D, Lansdorp P, Dokal I, Dror Y. Ataxia and pancytopenia caused by a mutation in TINF2. Hum Genet 2008; 124:

507-513.

50. Callewaert B, Su CT, Van Damme T, Vlummens P, Malfait F, Vanakker O, Schulz B, Mac Neal M, Davis EC, Lee JG, Salhi A, Unger S, Heimdal K, De Almeida

S, Kornak U, Gaspar H, Bresson JL, Prescott K, Gosendi ME, Mansour S, Piérard GE, Madan-Khetarpal S, Sciurba FC, Symoens S, Coucke PJ, Van

Maldergem L, Urban Z, De Paepe A. Comprehensive clinical and molecular analysis of 12 families with type 1 recessive cutis laxa. Hum Mutat 2013;

34: 111-121.

51. Urban Z, Hucthagowder V, Schürmann N, Todorovic V, Zilberberg L, Choi J, Sens C, Brown CW, Clark RD, Holland KE, Marble M, Sakai LY, Dabovic B,

Rifkin DB, Davis EC. Mutations in LTBP4 cause a syndrome of impaired pulmonary, gastrointestinal, genitourinary, musculoskeletal, and dermal

development. Am J Hum Genet 2009; 85: 593-605.

52. Sawyer SL, Dicke F, Kirton A, Rajapkse T, Rebeyka IM, McInnes B, Parboosingh JS, Bernier FP. Longer term survival of a child with autosomal recessive

cutis laxa due to a mutation in FBLN4. Am J Med Genet A 2013; 161a: 1148-1153.

53. Loeys B, De Paepe A, Urban Z. EFEMP2-Related Cutis Laxa. In: Adam MP, Ardinger HH, Pagon RA, Wallace SE, Bean LJH, Mirzaa G, Amemiya A, editors.

GeneReviews(®). Seattle (WA): University of Washington, Seattle Copyright © 1993-2021, University of Washington, Seattle. GeneReviews is a

registered trademark of the University of Washington, Seattle. All rights reserved.; 1993.

54. Rodriguez-Revenga L, Iranzo P, Badenas C, Puig S, Carrió A, Milà M. A novel elastin gene mutation resulting in an autosomal dominant form of cutis laxa.

Arch Dermatol 2004; 140: 1135-1139.

55. Urban Z, Gao J, Pope FM, Davis EC. Autosomal dominant cutis laxa with severe lung disease: synthesis and matrix deposition of mutant tropoelastin. J

Invest Dermatol 2005; 124: 1193-1199.

56. Duz MB, Kirat E, Coucke PJ, Koparir E, Gezdirici A, Paepe A, Callewaert B, Seven M. A novel case of autosomal dominant cutis laxa in a consanguineous

family: report and literature review. Clin Dysmorphol 2017; 26: 142-147.

57. Tekedereli I, Demiral E, Gokce IK, Esener Z, Camtosun E, Akinci A. Autosomal recessive cutis laxa: a novel mutation in the FBLN5 gene in a family. Clin

Dysmorphol 2019; 28: 63-65.

58. Milner JD, Vogel TP, Forbes L, Ma CA, Stray-Pedersen A, Niemela JE, Lyons JJ, Engelhardt KR, Zhang Y, Topcagic N, Roberson ED, Matthews H, Verbsky

JW, Dasu T, Vargas-Hernandez A, Varghese N, McClain KL, Karam LB, Nahmod K, Makedonas G, Mace EM, Sorte HS, Perminow G, Rao VK, O'Connell

MP, Price S, Su HC, Butrick M, McElwee J, Hughes JD, Willet J, Swan D, Xu Y, Santibanez-Koref M, Slowik V, Dinwiddie DL, Ciaccio CE, Saunders CJ,

Septer S, Kingsmore SF, White AJ, Cant AJ, Hambleton S, Cooper MA. Early-onset lymphoproliferation and autoimmunity caused by germline STAT3

gain-of-function mutations. Blood 2015; 125: 591-599.

59. Heimall J, Davis J, Shaw PA, Hsu AP, Gu W, Welch P, Holland SM, Freeman AF. Paucity of genotype-phenotype correlations in STAT3 mutation positive

Hyper IgE Syndrome (HIES). Clin Immunol 2011; 139: 75-84.

60. Al-Shaikhly T, Ochs HD. Hyper IgE syndromes: clinical and molecular characteristics. Immunol Cell Biol 2019; 97: 368-379.

61. Zhang Q, Davis JC, Lamborn IT, Freeman AF, Jing H, Favreau AJ, Matthews HF, Davis J, Turner ML, Uzel G, Holland SM, Su HC. Combined

immunodeficiency associated with DOCK8 mutations. N Engl J Med 2009; 361: 2046-2055.

62. Engelhardt KR, McGhee S, Winkler S, Sassi A, Woellner C, Lopez-Herrera G, Chen A, Kim HS, Lloret MG, Schulze I, Ehl S, Thiel J, Pfeifer D, Veelken H,

Niehues T, Siepermann K, Weinspach S, Reisli I, Keles S, Genel F, Kutukculer N, Camcioğlu Y, Somer A, Karakoc-Aydiner E, Barlan I, Gennery A, Metin

A, Degerliyurt A, Pietrogrande MC, Yeganeh M, Baz Z, Al-Tamemi S, Klein C, Puck JM, Holland SM, McCabe ER, Grimbacher B, Chatila TA. Large

deletions and point mutations involving the dedicator of cytokinesis 8 (DOCK8) in the autosomal-recessive form of hyper-IgE syndrome. J Allergy

Clin Immunol 2009; 124: 1289-1302.e1284.

63. Engelhardt KR, Gertz ME, Keles S, Schäffer AA, Sigmund EC, Glocker C, Saghafi S, Pourpak Z, Ceja R, Sassi A, Graham LE, Massaad MJ, Mellouli F, Ben-

Mustapha I, Khemiri M, Kilic SS, Etzioni A, Freeman AF, Thiel J, Schulze I, Al-Herz W, Metin A, Sanal Ö, Tezcan I, Yeganeh M, Niehues T, Dueckers G,

Weinspach S, Patiroglu T, Unal E, Dasouki M, Yilmaz M, Genel F, Aytekin C, Kutukculer N, Somer A, Kilic M, Reisli I, Camcioglu Y, Gennery AR, Cant

AJ, Jones A, Gaspar BH, Arkwright PD, Pietrogrande MC, Baz Z, Al-Tamemi S, Lougaris V, Lefranc G, Megarbane A, Boutros J, Galal N, Bejaoui M,

Barbouche MR, Geha RS, Chatila TA, Grimbacher B. The extended clinical phenotype of 64 patients with dedicator of cytokinesis 8 deficiency. J

Allergy Clin Immunol 2015; 136: 402-412.

64. Béziat V, Li J, Lin JX, Ma CS, Li P, Bousfiha A, Pellier I, Zoghi S, Baris S, Keles S, Gray P, Du N, Wang Y, Zerbib Y, Lévy R, Leclercq T, About F, Lim AI, Rao G,

Payne K, Pelham SJ, Avery DT, Deenick EK, Pillay B, Chou J, Guery R, Belkadi A, Guérin A, Migaud M, Rattina V, Ailal F, Benhsaien I, Bouaziz M, Habib

T, Chaussabel D, Marr N, El-Benna J, Grimbacher B, Wargon O, Bustamante J, Boisson B, Müller-Fleckenstein I, Fleckenstein B, Chandesris MO,

Titeux M, Fraitag S, Alyanakian MA, Leruez-Ville M, Picard C, Meyts I, Di Santo JP, Hovnanian A, Somer A, Ozen A, Rezaei N, Chatila TA, Abel L,

Leonard WJ, Tangye SG, Puel A, Casanova JL. A recessive form of hyper-IgE syndrome by disruption of ZNF341-dependent STAT3 transcription and

activity. Sci Immunol 2018; 3.

65. Frey-Jakobs S, Hartberger JM, Fliegauf M, Bossen C, Wehmeyer ML, Neubauer JC, Bulashevska A, Proietti M, Fröbel P, Nöltner C, Yang L, Rojas-Restrepo

J, Langer N, Winzer S, Engelhardt KR, Glocker C, Pfeifer D, Klein A, Schäffer AA, Lagovsky I, Lachover-Roth I, Béziat V, Puel A, Casanova JL,

Fleckenstein B, Weidinger S, Kilic SS, Garty BZ, Etzioni A, Grimbacher B. ZNF341 controls STAT3 expression and thereby immunocompetence. Sci

Immunol 2018; 3.

66. Sassi A, Lazaroski S, Wu G, Haslam SM, Fliegauf M, Mellouli F, Patiroglu T, Unal E, Ozdemir MA, Jouhadi Z, Khadir K, Ben-Khemis L, Ben-Ali M, Ben-

Mustapha I, Borchani L, Pfeifer D, Jakob T, Khemiri M, Asplund AC, Gustafsson MO, Lundin KE, Falk-Sörqvist E, Moens LN, Gungor HE, Engelhardt KR, Dziadzio M, Stauss H, Fleckenstein B, Meier R, Prayitno K, Maul-Pavicic A, Schaffer S, Rakhmanov M, Henneke P, Kraus H, Eibel H, Kölsch U, Nadifi S,

Nilsson M, Bejaoui M, Schäffer AA, Smith CI, Dell A, Barbouche MR, Grimbacher B. Hypomorphic homozygous mutations in phosphoglucomutase 3

(PGM3) impair immunity and increase serum IgE levels. J Allergy Clin Immunol 2014; 133: 1410-1419, 1419.e1411-1413.

67. Dadi H, Jones TA, Merico D, Sharfe N, Ovadia A, Schejter Y, Reid B, Sun M, Vong L, Atkinson A, Lavi S, Pomerantz JL, Roifman CM. Combined

immunodeficiency and atopy caused by a dominant negative mutation in caspase activation and recruitment domain family member 11 (CARD11). J

Allergy Clin Immunol 2018; 141: 1818-1830.e1812.

68. Breuer O, Daum H, Cohen-Cymberknoh M, Unger S, Shoseyov D, Stepensky P, Keller B, Warnatz K, Kerem E. Autosomal dominant gain of function STAT1

mutation and severe bronchiectasis. Respir Med 2017; 126: 39-45.

69. Aldave Becerra JC, Cachay Rojas E. A 3-Year-Old Girl with Recurrent Infections and Autoimmunity due to a STAT1 Gain-of-Function Mutation: The

Expanding Clinical Presentation of Primary Immunodeficiencies. Front Pediatr 2017; 5: 55.

70. Baris S, Alroqi F, Kiykim A, Karakoc-Aydiner E, Ogulur I, Ozen A, Charbonnier LM, Bakır M, Boztug K, Chatila TA, Barlan IB. Severe Early-Onset Combined

Immunodeficiency due to Heterozygous Gain-of-Function Mutations in STAT1. J Clin Immunol 2016; 36: 641-648.

71. Toubiana J, Okada S, Hiller J, Oleastro M, Lagos Gomez M, Aldave Becerra JC, Ouachée-Chardin M, Fouyssac F, Girisha KM, Etzioni A, Van Montfrans J,

Camcioglu Y, Kerns LA, Belohradsky B, Blanche S, Bousfiha A, Rodriguez-Gallego C, Meyts I, Kisand K, Reichenbach J, Renner ED, Rosenzweig S,

Grimbacher B, van de Veerdonk FL, Traidl-Hoffmann C, Picard C, Marodi L, Morio T, Kobayashi M, Lilic D, Milner JD, Holland S, Casanova JL, Puel A.

Heterozygous STAT1 gain-of-function mutations underlie an unexpectedly broad clinical phenotype. Blood 2016; 127: 3154-3164.

72. Vece TJ, Watkin LB, Nicholas S, Canter D, Braun MC, Guillerman RP, Eldin KW, Bertolet G, McKinley S, de Guzman M, Forbes L, Chinn I, Orange JS. Copa

Syndrome: a Novel Autosomal Dominant Immune Dysregulatory Disease. J Clin Immunol 2016; 36: 377-387.

73. Taveira-DaSilva AM, Markello TC, Kleiner DE, Jones AM, Groden C, Macnamara E, Yokoyama T, Gahl WA, Gochuico BR, Moss J. Expanding the phenotype

of COPA syndrome: a kindred with typical and atypical features. J Med Genet 2019; 56: 778-782.

74. Cho K, Yamada M, Agematsu K, Kanegane H, Miyake N, Ueki M, Akimoto T, Kobayashi N, Ikemoto S, Tanino M, Fujita A, Hayasaka I, Miyamoto S, Tanaka-

Kubota M, Nakata K, Shiina M, Ogata K, Minakami H, Matsumoto N, Ariga T. Heterozygous Mutations in OAS1 Cause Infantile-Onset Pulmonary

Alveolar Proteinosis with Hypogammaglobulinemia. Am J Hum Genet 2018; 102: 480-486.

75. Collin M, Dickinson R, Bigley V. Haematopoietic and immune defects associated with GATA2 mutation. Br J Haematol 2015; 169: 173-187.

76. Spinner MA, Sanchez LA, Hsu AP, Shaw PA, Zerbe CS, Calvo KR, Arthur DC, Gu W, Gould CM, Brewer CC, Cowen EW, Freeman AF, Olivier KN, Uzel G,

Zelazny AM, Daub JR, Spalding CD, Claypool RJ, Giri NK, Alter BP, Mace EM, Orange JS, Cuellar-Rodriguez J, Hickstein DD, Holland SM. GATA2

deficiency: a protean disorder of hematopoiesis, lymphatics, and immunity. Blood 2014; 123: 809-821.

77. Lo B, Zhang K, Lu W, Zheng L, Zhang Q, Kanellopoulou C, Zhang Y, Liu Z, Fritz JM, Marsh R, Husami A, Kissell D, Nortman S, Chaturvedi V, Haines H, Young

LR, Mo J, Filipovich AH, Bleesing JJ, Mustillo P, Stephens M, Rueda CM, Chougnet CA, Hoebe K, McElwee J, Hughes JD, Karakoc-Aydiner E, Matthews

HF, Price S, Su HC, Rao VK, Lenardo MJ, Jordan MB. AUTOIMMUNE DISEASE. Patients with LRBA deficiency show CTLA4 loss and immune

dysregulation responsive to abatacept therapy. Science 2015; 349: 436-440.

78. Azizi G, Abolhassani H, Mahdaviani SA, Chavoshzadeh Z, Eshghi P, Yazdani R, Kiaee F, Shaghaghi M, Mohammadi J, Rezaei N, Hammarström L,

Aghamohammadi A. Clinical, immunologic, molecular analyses and outcomes of iranian patients with LRBA deficiency: A longitudinal study. Pediatr

Allergy Immunol 2017; 28: 478-484.

79. Eren Akarcan S, Edeer Karaca N, Aksu G, Aykut A, Yilmaz Karapinar D, Cetin F, Aydinok Y, Azarsiz E, Gambineri E, Cogulu O, Ulusoy Severcan E, Alper H,

Kutukculer N. Two male siblings with a novel LRBA mutation presenting with different findings of IPEX syndrome. JMM Case Rep 2018; 5: e005167.

80. Gámez-Díaz L, August D, Stepensky P, Revel-Vilk S, Seidel MG, Noriko M, Morio T, Worth AJJ, Blessing J, Van de Veerdonk F, Feuchtinger T, Kanariou M,

Schmitt-Graeff A, Jung S, Seneviratne S, Burns S, Belohradsky BH, Rezaei N, Bakhtiar S, Speckmann C, Jordan M, Grimbacher B. The extended

phenotype of LPS-responsive beige-like anchor protein (LRBA) deficiency. J Allergy Clin Immunol 2016; 137: 223-230.

81. Kostel Bal S, Haskologlu S, Serwas NK, Islamoglu C, Aytekin C, Kendirli T, Kuloglu Z, Yavuz G, Dalgic B, Siklar Z, Kansu A, Ensari A, Boztug K, Dogu F,

Ikinciogullari A. Multiple Presentations of LRBA Deficiency: a Single-Center Experience. J Clin Immunol 2017; 37: 790-800.

82. Suzuki T, Sakagami T, Young LR, Carey BC, Wood RE, Luisetti M, Wert SE, Rubin BK, Kevill K, Chalk C, Whitsett JA, Stevens C, Nogee LM, Campo I, Trapnell

BC. Hereditary pulmonary alveolar proteinosis: pathogenesis, presentation, diagnosis, and therapy. Am J Respir Crit Care Med 2010; 182: 1292-

1304.

83. Hildebrandt J, Yalcin E, Bresser HG, Cinel G, Gappa M, Haghighi A, Kiper N, Khalilzadeh S, Reiter K, Sayer J, Schwerk N, Sibbersen A, Van Daele S, Nübling

G, Lohse P, Griese M. Characterization of CSF2RA mutation related juvenile pulmonary alveolar proteinosis. Orphanet J Rare Dis 2014; 9: 171.

84. Suzuki T, Maranda B, Sakagami T, Catellier P, Couture CY, Carey BC, Chalk C, Trapnell BC. Hereditary pulmonary alveolar proteinosis caused by recessive

CSF2RB mutations. Eur Respir J 2011; 37: 201-204.

85. Liu Y, Jesus AA, Marrero B, Yang D, Ramsey SE, Sanchez GAM, Tenbrock K, Wittkowski H, Jones OY, Kuehn HS, Lee CR, DiMattia MA, Cowen EW,

Gonzalez B, Palmer I, DiGiovanna JJ, Biancotto A, Kim H, Tsai WL, Trier AM, Huang Y, Stone DL, Hill S, Kim HJ, St Hilaire C, Gurprasad S, Plass N,

Chapelle D, Horkayne-Szakaly I, Foell D, Barysenka A, Candotti F, Holland SM, Hughes JD, Mehmet H, Issekutz AC, Raffeld M, McElwee J, Fontana JR,

Minniti CP, Moir S, Kastner DL, Gadina M, Steven AC, Wingfield PT, Brooks SR, Rosenzweig SD, Fleisher TA, Deng Z, Boehm M, Paller AS, Goldbach-

Mansky R. Activated STING in a vascular and pulmonary syndrome. N Engl J Med 2014; 371: 507-518.

86. Picard C, Thouvenin G, Kannengiesser C, Dubus JC, Jeremiah N, Rieux-Laucat F, Crestani B, Belot A, Thivolet-Béjui F, Secq V, Ménard C, Reynaud-Gaubert

M, Reix P. Severe Pulmonary Fibrosis as the First Manifestation of Interferonopathy (TMEM173 Mutation). Chest 2016; 150: e65-71.

87. Melki I, Rose Y, Uggenti C, Van Eyck L, Frémond ML, Kitabayashi N, Rice GI, Jenkinson EM, Boulai A, Jeremiah N, Gattorno M, Volpi S, Sacco O,

Terheggen-Lagro SWJ, Tiddens H, Meyts I, Morren MA, De Haes P, Wouters C, Legius E, Corveleyn A, Rieux-Laucat F, Bodemer C, Callebaut I, Rodero

MP, Crow YJ. Disease-associated mutations identify a novel region in human STING necessary for the control of type I interferon signaling. J Allergy

Clin Immunol 2017; 140: 543-552.e545.

88. Wangberg H, Wigby K, Jones MC. A novel autosomal dominant mutation in SOX18 resulting in a fatal case of hypotrichosis-lymphedema-telangiectasia

syndrome. Am J Med Genet A 2018; 176: 2824-2828.

89. Coulie R, Niyazov DM, Gambello MJ, Fastré E, Brouillard P, Vikkula M. Hypotrichosis-lymphedema-telangiectasia syndrome: Report of ileal atresia

associated with a SOX18 de novo pathogenic variant and review of the phenotypic spectrum. Am J Med Genet A 2021; 185: 2153-2159.

90. Valimahamed-Mitha S, Berteloot L, Ducoin H, Ottolenghi C, de Lonlay P, de Blic J. Lung involvement in children with lysinuric protein intolerance. J

Inherit Metab Dis 2015; 38: 257-263.

91. Mauhin W, Habarou F, Gobin S, Servais A, Brassier A, Grisel C, Roda C, Pinto G, Moshous D, Ghalim F, Krug P, Deltour N, Pontoizeau C, Dubois S, Assoun

M, Galmiche L, Bonnefont JP, Ottolenghi C, de Blic J, Arnoux JB, de Lonlay P. Update on Lysinuric Protein Intolerance, a Multi-faceted Disease

Retrospective cohort analysis from birth to adulthood. Orphanet J Rare Dis 2017; 12: 3.

92. Ramos MD, Trujillano D, Olivar R, Sotillo F, Ossowski S, Manzanares J, Costa J, Gartner S, Oliva C, Quintana E, Gonzalez MI, Vazquez C, Estivill X, Casals T.

Extensive sequence analysis of CFTR, SCNN1A, SCNN1B, SCNN1G and SERPINA1 suggests an oligogenic basis for cystic fibrosis-like phenotypes. Clin

Genet 2014; 86: 91-95.

93. Azad AK, Rauh R, Vermeulen F, Jaspers M, Korbmacher J, Boissier B, Bassinet L, Fichou Y, des Georges M, Stanke F, De Boeck K, Dupont L, Balascáková

M, Hjelte L, Lebecque P, Radojkovic D, Castellani C, Schwartz M, Stuhrmann M, Schwarz M, Skalicka V, de Monestrol I, Girodon E, Férec C, Claustres

M, Tümmler B, Cassiman JJ, Korbmacher C, Cuppens H. Mutations in the amiloride-sensitive epithelial sodium channel in patients with cystic

fibrosis-like disease. Hum Mutat 2009; 30: 1093-1103.

94. Sheridan MB, Fong P, Groman JD, Conrad C, Flume P, Diaz R, Harris C, Knowles M, Cutting GR. Mutations in the beta-subunit of the epithelial Na+

channel in patients with a cystic fibrosis-like syndrome. Hum Mol Genet 2005; 14: 3493-3498.

95. Hayasaka I, Cho K, Akimoto T, Ikeda M, Uzuki Y, Yamada M, Nakata K, Furuta I, Ariga T, Minakami H. Genetic basis for childhood interstitial lung disease

among Japanese infants and children. Pediatr Res 2018; 83: 477-483.

96. Nattes E, Lejeune S, Carsin A, Borie R, Gibertini I, Balinotti J, Nathan N, Marchand-Adam S, Thumerelle C, Fauroux B, Bosdure E, Houdouin V, Delestrain

C, Louha M, Couderc R, De Becdelievre A, Fanen P, Funalot B, Crestani B, Deschildre A, Dubus JC, Epaud R. Heterogeneity of lung disease associated

with NK2 homeobox 1 mutations. Respir Med 2017; 129: 16-23.

97. Hamvas A, Deterding RR, Wert SE, White FV, Dishop MK, Alfano DN, Halbower AC, Planer B, Stephan MJ, Uchida DA, Williames LD, Rosenfeld JA, Lebel

RR, Young LR, Cole FS, Nogee LM. Heterogeneous pulmonary phenotypes associated with mutations in the thyroid transcription factor gene NKX2-1.

Chest 2013; 144: 794-804.

98. van Meel E, Wegner DJ, Cliften P, Willing MC, White FV, Kornfeld S, Cole FS. Rare recessive loss-of-function methionyl-tRNA synthetase mutations

presenting as a multi-organ phenotype. BMC Med Genet 2013; 14: 106.

99. Hadchouel A, Wieland T, Griese M, Baruffini E, Lorenz-Depiereux B, Enaud L, Graf E, Dubus JC, Halioui-Louhaichi S, Coulomb A, Delacourt C, Eckstein G,

Zarbock R, Schwarzmayr T, Cartault F, Meitinger T, Lodi T, de Blic J, Strom TM. Biallelic Mutations of Methionyl-tRNA Synthetase Cause a Specific

Type of Pulmonary Alveolar Proteinosis Prevalent on Réunion Island. Am J Hum Genet 2015; 96: 826-831.

100. Abuduxikuer K, Feng JY, Lu Y, Xie XB, Chen L, Wang JS. Novel methionyl-tRNA synthetase gene variants/phenotypes in interstitial lung and liver disease:

A case report and review of literature. World J Gastroenterol 2018; 24: 4208-4216.

101. Karolak JA, Vincent M, Deutsch G, Gambin T, Cogné B, Pichon O, Vetrini F, Mefford HC, Dines JN, Golden-Grant K, Dipple K, Freed AS, Leppig KA, Dishop

M, Mowat D, Bennetts B, Gifford AJ, Weber MA, Lee AF, Boerkoel CF, Bartell TM, Ward-Melver C, Besnard T, Petit F, Bache I, Tümer Z, Denis-

Musquer M, Joubert M, Martinovic J, Bénéteau C, Molin A, Carles D, André G, Bieth E, Chassaing N, Devisme L, Chalabreysse L, Pasquier L, Secq V,

Don M, Orsaria M, Missirian C, Mortreux J, Sanlaville D, Pons L, Küry S, Bézieau S, Liet JM, Joram N, Bihouée T, Scott DA, Brown CW, Scaglia F, Tsai

AC, Grange DK, Phillips JA, 3rd, Pfotenhauer JP, Jhangiani SN, Gonzaga-Jauregui CG, Chung WK, Schauer GM, Lipson MH, Mercer CL, van Haeringen

A, Liu Q, Popek E, Coban Akdemir ZH, Lupski JR, Szafranski P, Isidor B, Le Caignec C, Stankiewicz P. Complex Compound Inheritance of Lethal Lung

Developmental Disorders Due to Disruption of the TBX-FGF Pathway. Am J Hum Genet 2019; 104: 213-228.

102. Klar J, Blomstrand P, Brunmark C, Badhai J, Håkansson HF, Brange CS, Bergendal B, Dahl N. Fibroblast growth factor 10 haploinsufficiency causes

chronic obstructive pulmonary disease. J Med Genet 2011; 48: 705-709.

103. Sasaki E, Byrne AT, Phelan E, Cox DW, Reardon W. A review of filamin A mutations and associated interstitial lung disease. Eur J Pediatr 2019; 178: 121-

129.

104. Pelizzo G, Collura M, Puglisi A, Pappalardo MP, Agolini E, Novelli A, Piccione M, Cacace C, Bussani R, Corsello G, Calcaterra V. Congenital

emphysematous lung disease associated with a novel Filamin A mutation. Case report and literature review. BMC Pediatr 2019; 19: 86.

105. Shah AS, Black ED, Simon DM, Gambello MJ, Garber KB, Iannucci GJ, Riedesel EL, Kasi AS. Heterogeneous Pulmonary Phenotypes in Filamin A Mutation-

Related Lung Disease. Pediatr Allergy Immunol Pulmonol 2021; 34: 7-14.

106. de Bruyn G, Casaer A, Devolder K, Van Acker G, Logghe H, Devriendt K, Cornette L. Hydrops fetalis and pulmonary lymphangiectasia due to FOXC2

mutation: an autosomal dominant hereditary lymphedema syndrome with variable expression. Eur J Pediatr 2012; 171: 447-450.

107. Sargent C, Bauer J, Khalil M, Filmore P, Bernas M, Witte M, Pearson MP, Erickson RP. A five generation family with a novel mutation in FOXC2 and

lymphedema worsening to hydrops in the youngest generation. Am J Med Genet A 2014; 164a: 2802-2807.

108. Myers A, du Souich C, Yang CL, Borovik L, Mwenifumbo J, Rupps R, Study C, Lehman A, Boerkoel CF. FOXP1 haploinsufficiency: Phenotypes beyond

behavior and intellectual disability? Am J Med Genet A 2017; 173: 3172-3181.

109. Nicolaou N, Margadant C, Kevelam SH, Lilien MR, Oosterveld MJ, Kreft M, van Eerde AM, Pfundt R, Terhal PA, van der Zwaag B, Nikkels PG, Sachs N,

Goldschmeding R, Knoers NV, Renkema KY, Sonnenberg A. Gain of glycosylation in integrin α3 causes lung disease and nephrotic syndrome. J Clin

Invest 2012; 122: 4375-4387.

110. He Y, Balasubramanian M, Humphreys N, Waruiru C, Brauner M, Kohlhase J, O'Reilly R, Has C. Intronic ITGA3 Mutation Impacts Splicing Regulation and

Causes Interstitial Lung Disease, Nephrotic Syndrome, and Epidermolysis Bullosa. J Invest Dermatol 2016; 136: 1056-1059.

111. Has C, Spartà G, Kiritsi D, Weibel L, Moeller A, Vega-Warner V, Waters A, He Y, Anikster Y, Esser P, Straub BK, Hausser I, Bockenhauer D, Dekel B,

Hildebrandt F, Bruckner-Tuderman L, Laube GF. Integrin α3 mutations with kidney, lung, and skin disease. N Engl J Med 2012; 366: 1508-1514.

112. Yalcin EG, He Y, Orhan D, Pazzagli C, Emiralioglu N, Has C. Crucial role of posttranslational modifications of integrin α3 in interstitial lung disease and

nephrotic syndrome. Hum Mol Genet 2015; 24: 3679-3688.

113. Colombo EA, Spaccini L, Volpi L, Negri G, Cittaro D, Lazarevic D, Zirpoli S, Farolfi A, Gervasini C, Cubellis MV, Larizza L. Viable phenotype of ILNEB

syndrome without nephrotic impairment in siblings heterozygous for unreported integrin alpha3 mutations. Orphanet J Rare Dis 2016; 11: 136.

114. Barnett CP, Nataren NJ, Klingler-Hoffmann M, Schwarz Q, Chong CE, Lee YK, Bruno DL, Lipsett J, McPhee AJ, Schreiber AW, Feng J, Hahn CN, Scott HS.

Ectrodactyly and Lethal Pulmonary Acinar Dysplasia Associated with Homozygous FGFR2 Mutations Identified by Exome Sequencing. Hum Mutat

2016; 37: 955-963.

115. Cutting GR. Cystic fibrosis genetics: from molecular understanding to clinical application. Nat Rev Genet 2015; 16: 45-56.

116. Deignan JL, Astbury C, Cutting GR, Del Gaudio D, Gregg AR, Grody WW, Monaghan KG, Richards S. CFTR variant testing: a technical standard of the

American College of Medical Genetics and Genomics (ACMG). Genet Med 2020; 22: 1288-1295.

117. Elborn JS. Cystic fibrosis. Lancet 2016; 388: 2519-2531.

118. Spinnato P, Facchini G, Tetta C, Lotrecchiano L, Colangeli M, Bazzocchi A, Albisinni U, Cutrera R, Tomà P, Bartoloni A. Neurofibromatosis type-1-

associated diffuse lung disease in children. Pediatr Pulmonol 2019; 54: 1760-1764.

119. Tognato E, Perona A, Aronica A, Bertola A, Cimminelli L, De Vecchi S, Eshraghy MR, Loperfido B, Vivenza C, Manzoni P. Neonatal Marfan Syndrome. Am

J Perinatol 2019; 36: S74-s76.

120. Dietz H. Marfan Syndrome. In: Adam MP, Ardinger HH, Pagon RA, Wallace SE, Bean LJH, Mirzaa G, Amemiya A, editors. GeneReviews(®). Seattle (WA):

University of Washington, Seattle Copyright © 1993-2021, University of Washington, Seattle. GeneReviews is a registered trademark of the

University of Washington, Seattle. All rights reserved.; 1993.

121. Lo IF, Brewer C, Shannon N, Shorto J, Tang B, Black G, Soo MT, Ng DK, Lam ST, Kerr B. Severe neonatal manifestations of Costello syndrome. J Med

Genet 2008; 45: 167-171.

122. O'Shea J, Lynch SA, Macken S. A case of persistent pulmonary hypertension in a newborn with Costello syndrome. Clin Dysmorphol 2008; 17: 287-288.

123. Gomez-Ospina N, Kuo C, Ananth AL, Myers A, Brennan ML, Stevenson DA, Bernstein JA, Hudgins L. Respiratory system involvement in Costello

syndrome. Am J Med Genet A 2016; 170: 1849-1857.
